# CO_2_ Capture by Porous Polymeric Sorbents: From New *cis‑*/*trans*-Oxovanadium(IV) Catalyst to Functional Materials

**DOI:** 10.1021/acs.jpcb.5c01060

**Published:** 2025-06-13

**Authors:** Kacper Pobłocki, Marzena Białek, Katarzyna N. Jarzembska, Mateusz A. Baluk, Radosław Kamiński, Przemysław Rybiński, Krzysztof Matus, Joanna Drzeżdżon, Barbara Gawdzik, Dagmara Jacewicz

**Affiliations:** † Department of Environmental Technology, Faculty of Chemistry, 49646 University of Gdansk Wita Stwosza 63, 80-308 Gdansk, Poland; ‡ Department of Chemical Technology and Polymer Chemistry, Institute of Chemistry, Institute of Chemistry, University of Opole, Oleska 48, 45-052 Opole, Poland; § Department of Chemistry, University of Warsaw, Żwirki i Wigury 101, 02-089 Warsaw, Poland; ∥ Institute of Chemistry, Jan Kochanowski University, Uniwersytecka 7, 25-406 Kielce, Poland; ⊥ Materials Research Laboratory, 49569 Silesian University of Technology, Konarskiego 18A, 44-100 Gliwice, Poland

## Abstract

The challenge of ever-increasing greenhouse gas emissions correlates with intensive research into the development of new CO_2_ sorbents. The research aimed to propose a new generation of porous polymer sorbents that contribute to decarbonization. Among all oligomers and (co)­polymers, the 3-buten-2-ol oligomer showed the highest CO_2_ sorption properties (0.82 mmol g^–1^). The heat of adsorption of CO_2_ by oligo­(3-buten-2-ol) was equal to 8.51 kJ mol^–1^. Polyolefins and polar oligomers were synthesized using a new, innovative, and highly active oxovanadium­(IV) precatalyst. An easy, one-step method for the synthesis of *cis*/*trans*-[VO­(acac)_2_(3-ppy)] provides a unique coordination compound containing two molecules that are reciprocal geometric isomers with a spatial arrangement of acetylacetonate ligands (*cis* and *trans*). The complex shows very active catalytic properties in the polymerization reactions of olefins (83,400 kg_PE_ mol_V_^–1^ h^–1^) and polar monomers, for example, 2-propen-1-ol (1060 kg mol_V_^–1^ h^–1^). A physicochemical study was also conducted to determine the stability constant of the complex formation, log β_1210_ = 27.82.

## Graphical abstract

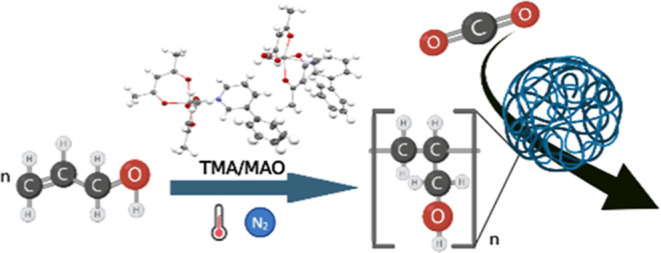

## 1 Introduction

The structure of a precatalyst, including the spherical hindrance and electronic factors, plays a key role in the synthesis of polymeric materials.^1,2^ Vanadium­(IV/V) precatalysts attract attention due to the possibility of synthesizing ultrahigh-molecular-weight polyethylene (with a controlled degree of dispersity) and uniform incorporation of higher olefins (1-octene or 1-hexene) into the polyolefin chain.^1,2^ Additionally, Dixit et al. showed that catalytic systems based on the vanadium­(III/IV) cation are more active in the reactions of anionic coordination polymerization of polar monomers (i.e., acrylonitrile) than those containing the titanium­(IV) cation.^3^ Nevertheless, vanadium­(III/IV) precatalysts usually do not show higher catalytic properties in comparison to chemical entities based on titanium­(IV) or chromium­(III) cations. One of the challenges is to develop kinetically stable catalytic systems based on vanadium­(IV/V) cations to prevent rapid deactivation and reduction to inactive forms of the vanadium­(II) cation.^4,5^ There are many studies devoted to exploring the catalytic activity of simple vanadium metal salts. However, many problems have been encountered, such as low thermal stability at room temperature, aggregation of polar activators, or adsorption of monomers, which correlate with reduced catalytic activity. Natta et al. discovered that not only metal cation halides are suitable precatalysts in the olefin polymerization reaction but also β-diketones were used as strongly chelating bidentate ligands in the complex.^6^ By creating a flat, six-membered ring, which stabilized the metal ion as the active center of the catalyst,^7^ V­(acac)_3_/Et_2_AlCl (acac = acetylacetonate anion) was used as a catalytic system in the synthesis of syndiotactic polypropylene. It was discovered that vanadium coordination compounds were thermally unstable because VCl_2_ precipitated already at room temperature.^6^ In turn, Kothandaraman and Devi presented the use of VO­(acac)_2_/AlEt_3_ and VO­(acac)_2_/Et_2_AlBr in the polymerization reaction of 1-octene.^8^ A high molecular weight of poly­(1-octene) was obtained, characterized by a low isotacticity (22–24%). The presented assumptions are based, among others, on the fact that due to the polar nature of the Et_2_AlBr activator, aggregation, and reduction of active sites on the catalyst may occur. Additionally, there is a possibility of the adsorption of monomers and reduced catalytic activity. The authors indicated a reduction in the tacticity of the obtained polymers resulting from the presence of oxygen in vanadium precatalysts. This effect can be attributed to the occurrence of the resonance structures. Therefore, the coordination of the monomer is weaker, which affects the growth of the macromolecule chain. VO­(acac)_2_ is more stable in air than simple inorganic vanadium salts, i.e., VCl_3_ VOCl_2_ and VOCl_3_, which are air sensitive.^9^

Due to the rapid deactivation of vanadium catalysts to inactive vanadium­(II) centers, there is a need to report protocols for the synthesis (Figure 1A) of kinetically stable coordination compounds of vanadium ions acting as precatalysts. Therefore, a coordination compound of oxovanadium­(IV) acetylacetonate with an electron-donating ligand, i.e., 3-phenylpyridine, was designed as *cis*/*trans*-[VO­(acac)_2_(3-ppy)] (**1**) (3-phenylpyridine = 3-ppy). So far, no one in the literature has used a catalytic system based on geometric isomers in the polymerization and oligomerization reactions of olefins. A research gap was filled through the easy, one-pot synthesis of a coordination compound in the form of both *cis* and *trans* geometric isomers. The synthesized polar oligomers were tested for the first time in terms of carbon dioxide and nitrogen sorption properties. Oligomers of 2-propen-1-ol, 2-chloro-2-propen-1-ol, 2,3-dibromo-2-propen-1-ol, and 3-buten-2-ol are a new generation of materials for N_2_ and CO_2_ sorption. Research shows that they constitute an innovative class of sorption materials. Our research provides a new perspective on the development of polyolefins and polar oligomers toward sustainable development. A comparison of the materials known in the literature in terms of CO_2_ sorption properties is given in Table 1.

**Figure 1:**
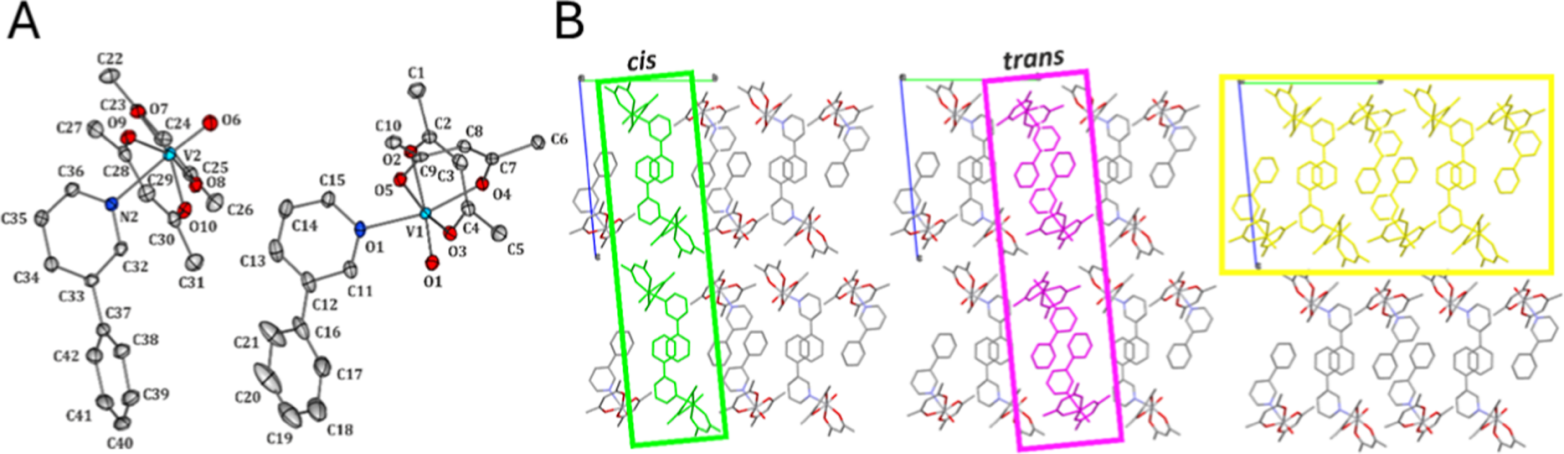
Molecular structure of the studied complex **1** [VO­(acac)_2_(3-ppy)] (*cis* and *trans* isomers comprising ASU) with the labeling scheme (atomic thermal motion represented as ellipsoids drawn at the 50% probability level; hydrogen atoms removed for clarity) (a). Crystal packing of 1 shown along the *X* axis: (010) layers (green) composed of *cis* molecules; (010) layers (magenta) composed of *trans* molecules; (001) layers (yellow) composed of both *cis* and *trans* molecules. Hydrogens atoms are omitted for clarity (b).

**Table 1:** Comparison of Materials Characterized by CO_2_ Sorption Properties^c^

materials	SA_BET_ (m^2^/g)	CO_2_ uptake at 1 bar and at 298 K (mmol/g)	heat of adsorption (*Q*_st_) (kJ/mol)	CO_2_/N_2_ selectivity at 298 K	references
oligo(3-buten-2-ol)^a^	12	0.82	8.51	22.4	this work
oligo(3-buten-2-ol)^b^	98	4.33			this work
TPPM (3D-triptycene and phenanthroline-based microporous polymer)	1120	1.85	23	20	^10^
TBMP-NH_2_ (3D-molecular building block triptycene)	866	1.23	33.95	17	^11^
TBPP-OH (3D-triptycene-containing hyper-cross-linked microporous polymer possessing hydroxyl groups)	838	2.77 (273 K)	32.9	18	^12^
nitrogen-doped carbons NDC-700 (biomass-derived carbons from Albizia procera leaves)	426	2.11		54	^13^
Jute-derived carbons	334	1.61		54	^14^
membrane HDPE		4.82 (303.15 K)			^15^
coal-tar-based hyper-cross-linked polymers (CTHP)	826	1.94	30.6	32.3	^16^
activated carbon	2829	2.92			^17^
polyester (1,3,5-benzenetricarbonyl trichloride and cyanuric acid)	11.2	0.163			^18^
TrzPOP-1 [microporous polymer based on 1,4-bis(4,6-diamino-*s*-triazin-2-yl)benzene and terepthaldehyde]	995	3.53	29	42.1	^19^
TrzPOP-2 [microporous polymer based on 1,4-bis(4,6-diamino-*s*-triazin-2-yl)benzene and 2,6-diformyl-4-methylphenol]	868	4.52	34	75.7	^19^
TrzPOP-3 [microporous polymer based on 1,4-bis(4,6-diamino-*s*-triazin-2-yl)benzene and diformylphloroglucinol]	772	5.09	37	94.5	^19^

a Outgassing temperature 60 °C.

b Outgassing temperature 100 °C.

c SA_BET_ = surface area BET.

## 2 Materials and Methods

### 2.1 Materials

VO­(acac)_2_, 3-phenylpyridine, 2-propen-1-ol, 2-chloro-2-propen-1-ol, 3-buten-2-ol, 2,3-dibromo-2-propen-1-ol, MAO (methylaluminoxane), and TMA (trimethylaluminum) were purchased from Merck. Argon (grade 5.0, Linde Gas), Et_2_AlCl (1.0 M, Sigma-Aldrich), and EtAlCl_2_ (1.0 M, Sigma-Aldrich) were used as received. Ethyl trichloroacetate (Acros Organics), methylene chloride (p.a., POCh), and 1-octene (99.9%, Thermo Scientific) were dried over molecular sieves 4A. Hexane (fraction from petroleum, Linegal) was dried by refluxing over sodium/benzophenone in a nitrogen atmosphere and then distilled before use. Ethylene (grade 3.5, Air Liquide) and nitrogen (Messer) were used after having been passed through a column with sodium metal supported on Al_2_O_3_.

### 2.2 X-ray Diffraction

Single-crystal X-ray measurement was performed on a Rigaku Oxford Diffraction SuperNova instrument equipped with a microfocus copper X-ray source (Cu Kα radiation, λ = 1.54184 Å). During the measurement, the crystal was maintained at 100 K with the use of an Oxford Cryosystems nitrogen gas-flow device. Unit-cell parameter determination and raw diffraction image processing were performed with the native diffractometer CRYSALISPRO software suite. Structure was solved using an intrinsic phasing method as implemented in the SHELXT program^20^ and refined with the JANA package^21^ within the independent atom model approximation. The hydrogen atoms were placed geometrically (*d*_C–H_ = 0.96 Å). The riding model for the hydrogen thermal motion parameters was applied (*U*_iso_^H^ = *x*·*U*_eq_^X^ where *x* = 1.2 for X = C). The original CIF files are also available from the Supporting Information or can be retrieved from the Cambridge Structural Database^22,23^ (deposition number: CCDC 2316951).

#### 2.2.1 Synthesis of the Coordination Complex Compound [VO­(acac)_2_(3-phenylpyridine)]

The synthesis method for obtaining a new coordination compound is as follows. To 30 mL of an aqueous solution of oxovanadium­(IV) acetylacetonate (1.0 mmol) was added 1 mmol of 3-phenylpyridine, in liquid form (Figure 1A). The mixture was then heated to reflux for 2 h using a stirred heating mantle. In the next step, the mixture was cooled and left in the refrigerator. After a few days, green crystals of compound **1** were obtained in a yield of 30.1%.

### 2.3 Polymerization of Ethylene

Polymerizations were carried out in a 500 mL Büchi glass autoclave equipped with a magnetic stirrer and heating–cooling jacket. The reactor was initially heated and purged with nitrogen, and the reaction temperature was set up (60 °C). The following chemical reagents were added to the reactor solvent (hexane, 100 mL), ethyl trichloroacetate (ETA), alkylaluminum compound, and a solution of the complex in CH_2_Cl_2_ in this order under an inert atmosphere, and finally ethylene was introduced. Each polymerization was conducted for 10 min under 5 or 3 bar of ethylene. The ethylene pressure and reactor temperature were kept constant during polymerization. Reaction was terminated by venting the reactor and transferring the obtained mixture to a dilute solution of hydrochloric acid in methanol. The polymer was filtered, adequately washed with methanol, and dried to constant weight under a vacuum.

### 2.4 Copolymerization of Ethylene with 1-Octene

The copolymerization was performed in the same manner as homopolymerization with the exception that 1-octene was added after solvent addition.

### 2.5 Oligomerization of Polar Monomers

The procedure was identical to that in previous works.^24^ First, coordination compound **1** was dissolved in anhydrous toluene (2 mL) in a 9 mL glass vial. Nitrogen was purged for 15 min to remove air. Then the activator was added (1–3 mL). In the next step, a polar monomer was added dropwise, i.e., 2-propen-1-ol, 2-chloro-2-propen-1-ol, 2,3-dibromo-2-propen-1-ol, and 3-buten-2-ol (3 mL). After 30 min, a sticky gel precipitated and was washed with 1 M HCl in methanol. The oligomer was dried to a constant weight in a vacuum oven for 12 h.

### 2.6 MALDI-TOF-MS Characterization of Polymeric Materials

The procedure is identical to that in previous studies.^25^ The MALDI-TOF-MS spectra were recorded on the Bruker Biflex III (Branch Überlingen, Germany). 2,5-Dihydroxybenzoic acid (DHB) was used as a matrix.

### 2.7 Thermal Analysis

Thermal analysis (TG) was performed on a NETZSCH TG 209 instrument in an argon atmosphere in the temperature range from 0 to 1000 °C. The mass of samples subjected to thermal analysis was about 5 mg.

### 2.8 Differential Scanning Calorimetry

Differential scanning calorimetry (DSC) studies of oligomers were performed using the equipment from Mettler Toledo in the range from −150 to 500 °C at a heating rate of 10 °C·min^–1^ in an inert atmosphere. The sample for the DSC measurements was about 5 mg.

DSC measurements for polyethylene and copolymers were conducted using a DSC1 Mettler Toledo instrument at the heating rate of 10 °C/min. The melting temperatures (*T*_m_) and melting enthalpies (Δ*H*) were obtained from the second heating cycle. The degree of crystallinity (*C*) was calculated using the following equation: *C* (%) = Δ*H*·(100/290 J/g).^26^

### 2.9 Characterization of Polyethylene and Copolymers by FTIR and NMR Spectroscopy

The FTIR spectra of polyethylene and copolymers were recorded in the range from 4000 to 400 cm^–1^ with a 2 cm^–1^ resolution on the Nicole Nexus 2002 FTIR spectrometer. The samples were prepared as a pellet made of polymer powder and KBr. Comonomer incorporation was calculated according to the method described in ref 27 based on absorption bands characteristic for symmetrical in-plane bending deformations of CH_3_ (1379 cm^–1^) and CH_2_ (1369 cm^–1^) groups (scissoring δ_s_ vibrations) using a calibration curve prepared for ethylene/1-octene copolymers. ^13^C NMR spectra of copolymers were taken on a Bruker Ultrashield 400 spectrometer. Analyses were performed at 120 °C using o-dichlorobenzene-d4 as a solvent. Chemical shifts were referenced internally to the major backbone methylene carbon resonance, which was taken to be 30.00 ppm.

### 2.10 Surface Morphology Characterization of Polymeric Materials

The surface morphology of the synthesized materials was examined by scanning electron microscopy (SEM) using a JEOL JSM-7610F instrument and transmission electron microscopy (TEM) was performed with the FEI S/TEM TITAN 80-300 electron microscope. This instrument was set to an operating voltage of 300 kV and featured a Schottky-type field emission gun. It also includes a CETCOR Cs-probe corrector by the CEOS Company and an Energy-Dispersive X-ray Spectroscopy (EDS) detector.

### 2.11 Sorption Characterization of Polymeric Materials

Sorption tests were performed on the preheated material at 60 °C or 100 °C for 5 h under vacuum. Sorption properties were investigated using a Micro 200 C analyzer (3P Instruments). The results, Brunauer–Emmett–Teller (BET) and Langmuir surface area data, N_2_ and CO_2_ adsorption and desorption isotherms, pore size, and volume, were recorded by PES software. Pore size distribution was calculated using the Barrett–Joyner–Halenda (BJH) method (by PAS software), according to the Halsey equation (eq 1). The BET specific surface area was studied at 77 K (in liquid nitrogen), while N_2_ and CO_2_ capacity was studied at 298 K. The equation describing the calculation of selectivity is shown in eq 3. For the selected sample, CO_2_ sorption was studied between 223 and 298 K and the heat of adsorption was calculated using the Clausius–Clapeyron equation (eq 2)

Equation 1

1 $$t={\left(\frac{-1}{\ln\left(\frac{p}{p_{0}}\right)}\right)}^{1/3}$$

where *p*/*p*_0_—relative pressure, and *t*—the thickness of the layer of condensate adsorbed on the pore surface at a given point in the adsorption isotherm

Equation 2

2 $$\ln{\left(\frac{p1}{p2}\right)}_{\Theta }=\frac{-\Delta H_{ads}}{R}\left(\frac{1}{T1}-\frac{1}{T2}\right)$$

where Δ*H*_ads_—heat of adsorption, *p*1 and *p*2 are pressure of CO_2_ at temperature *T*1 and *T*2, respectively, and *R*—the ideal gas constant

Equation 3

3 $$S\frac{{CO}_{2}}{N_{2}}=\frac{q{CO}_{2}}{qN_{2}}$$

where *S*—selectivity, and *q*CO_2_/N_2_ is the amount of adsorbed gas (e.g., in mmol/g) under the same conditions (temperature, pressure).

### 2.12 Potentiometric Titrations

A 50 mM NaOH solution was used as the titrant in the experiment, which was dosed at 0.001 mL every 30 s. The pH electrode was calibrated during potentiometric measurements in accordance with IUPAC (International Union of Pure and Applied Chemistry) guidelines. For this purpose, buffer solutions with precisely defined pH were prepared. In addition, it was verified that the reference electrode, characterized by a standard potential, was properly filled with the electrolyte. The process of calibrating the potentiometric electrode was carried out at three different pH points (thermostat). The titrated solution contained VO^2+^ (1 mM), acac (2 mM), 3-ppy (1 mM), and HClO_4_ (5 mM). Hyperquad2008 software was used to fit the experimental curve to the theoretical curve and determine the log β value. The theoretical model was correlated to the experimental results to determine the stability constants of the complex. The model used is described in eqs 3 and 4.

Equations 3 and 4

pM+qL+rB+sH=MpLqBrHs

4 $$\beta_{pqrs}=\frac{\left[M_{p}L_{q}B_{r}H_{s}\right]}{{\left[M\right]}^{p}{\left[L\right]}^{q}{\left[B\right]}^{r}{\left[H\right]}^{s}}$$

where p, q, r, and s—stoichiometric coefficients for the reaction, M = VO^2+^, L = acetyloacetonate, B = 3-ppy, H = proton (Table 2).

**Table 2:** Log β_pqrs_ Values for the [VO­(acac)_2_(3-ppy)] Complex^a^

formed species	log β_pqrs_
ML_2_B	log β_1210_ = 27.82 (±0.10)
ML_2_	log β_1200_ = 23.24 (±0.45)
ML	log β_1100_ = 15.22 (±0.25)
MH_–4_	log β_100–4_ = −19.05 (±0.10)
LH_2_	log β_0102_ = 14.03 (±0.22)
LH	log β_0102_ = 9.77 (±0.04)
LH_–1_	log β_010–1_ = −8.85 (±0.03)

a Standard deviations are presented in parentheses, where M = VO^2+^, L = acac, and B = 3-ppy.

### 2.13 Conductometric Titrations

Conductometric titrations were carried out using a Cerko Lab apparatus equipped with a CD-201 conductivity cell (conductivity constant *k* = 0.096^–1^, HYDROMET), a magnetic stirrer, and a 5 mL Hamilton syringe. Electrode standardization was carried out using potassium chloride solutions purchased from Hamilton, with conductivities of 84 μS/cm, 147,9 μS/cm, and 200 μS/cm. Experiments were performed at 298.15 K, using a Lauda E100 circulating thermostat. A 50 mM NaOH solution was used as a titrant, which was dispensed at 30 s intervals. The titrated solution contained VO^2+^ (1 mM), acac (2 mM), and 3-ppy (1 mM).

## 3 Results

### 3.1 Crystal Structure

Complex 1 crystallizes in the triclinic *P* 1̅ space group with two molecules in the asymmetric unit (ASU) (Figure 1B). The crystalline structure was examined by XRD and further characterized by TGA, FT-IR (Figure 2A,B), UV–vis, and UV–vis DRS (Figures 1C and S38–40). The metal centers in both symmetry-independent complex molecules are coordinated via oxygen atoms by two bidentate acetylacetonate ligands, via a nitrogen atom with a 3-phenylpyridine moiety, and by an oxygen atom, which results in a distorted octahedral coordination geometry. Interestingly, the two molecules comprising ASU constitute mutual geometric isomers with a *cis* and *trans* spatial arrangement of the acac ligands. In the crystal structure, molecules of each isomer are organized in separate layers parallel to the (010) crystal plane and repeat in space alternately along the [010] crystallographic direction (Figure 1C). Alternatively, double molecular layers parallel to the (001) crystal plane can be distinguished. In this case, the center-of-symmetry-related *cis* and *trans* species interact one with another via rather distant π···π stacking interactions (*cis*/*cis*) and edge-to-face-like contacts (*trans*/*trans*) and via some other dispersive contacts between the adjacent phenylpyridyl moieties. Furthermore, the latter ligands form hydrogen-bond-like contacts with the acac fragments, i.e., the C35–H35···O3 and C14–H14···O10 interactions, and with oxygen ligands, namely, the C35–H35···O1, C40–H40···O1, and C14–H14···O6 interactions. In turn, the acac species, grouped together in-between the 3-phenylpyridyl regions, are also engaged in numerous C–H···O interactions with the neighboring acac species (O7···H6b and O9···H6b) and oxygen ligands (O6···H8, O6···H6a; O1···H22a, O1···H5a, O1···H40). The coordination compound oxovanadium­(IV) is a racemate; that is, it consists of 50% of the *cis* form and 50% of the *trans* form. Additional confirmation of the crystal structure are the FT-IR, UV–vis, and UV–vis DRS spectra and SEM images obtained (Figure S1–S4).

**Figure 2:**
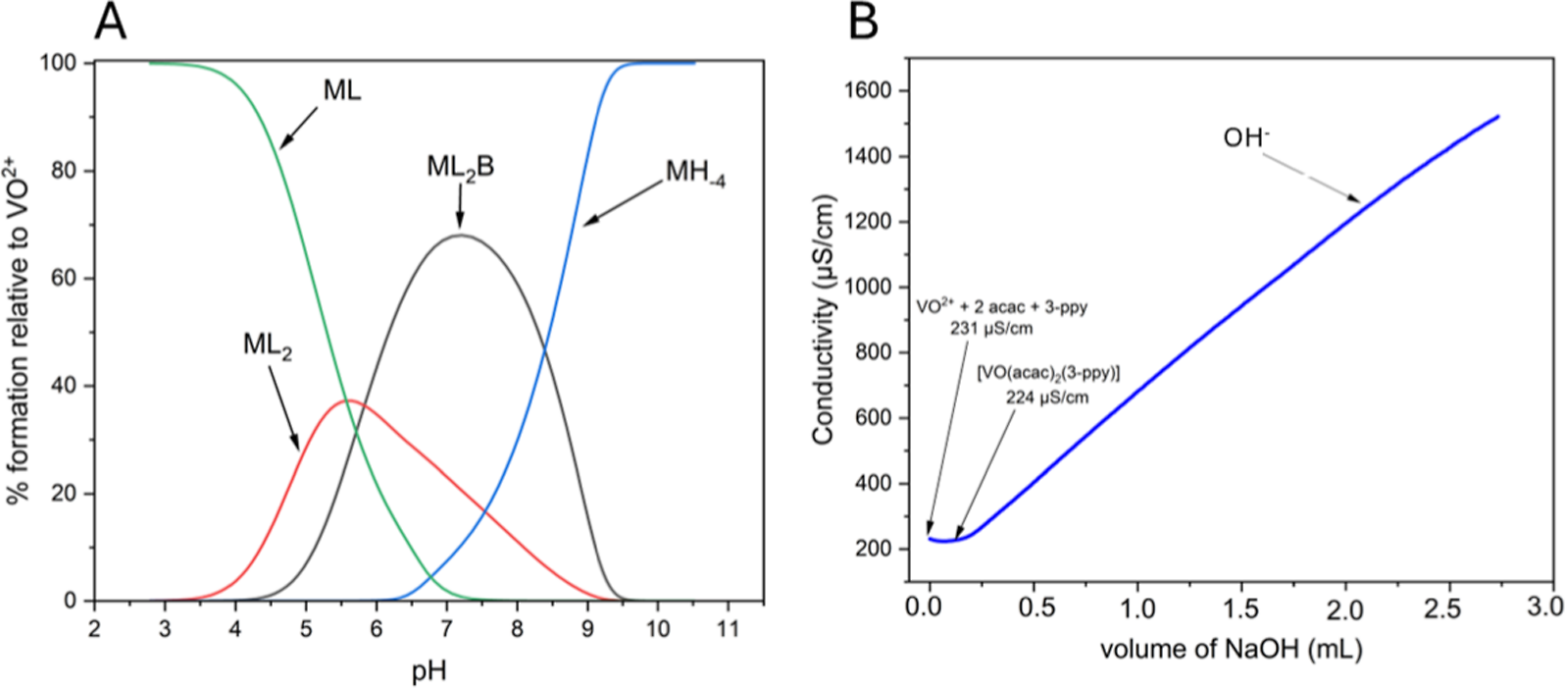
Concentration distribution curves of the complexes as a function of pH calculated based on the stability constants concerning *cis-*/*trans*-[VO­(acac)_2_(3-ppy)] formed in the system VO^2+^/acac/3-ppy (molar ratio 1:2:1) (a). The conductivity titration curves VO^2+^ ions with acac and 3-phenylpyridine (b).

### 3.2 Physicochemical Properties of *Cis*/*Trans*-[VO­(acac)_2_(3-ppy)]

There are several types of stability of coordination compounds, e.g., thermal, thermodynamic, and kinetic stability. A newly synthesized coordination compound decomposes in one step at 250 °C. The rate of this step is 7.5%/min (Figure S5). Potentiometric studies were performed to determine the formation constant of the *cis*/*trans*-[VO­(acac)_2_(3-ppy)] complex and determine its thermodynamic stability (Figure S6 and Table 2). The value of the formation of the complex is equal to log β_1210_ = 27.82. It was proved that the addition of 3-phenylpyridine increased the stability of the coordination compound. The value of the persistence constant of the protonated acac is close to the literature values and is ∼9.^28^ In addition, it was shown that the highest concentration of the newly synthesized coordination compound oxovanadium­(IV) predominates at pH 7–7.5. As the pH increases to about 8, the concentration of hydroxide complex MH_–4_ increases. In turn, the concentration of compound VO­(acac)_2_ predominates at a pH equal to 5.5 and is about 38% in aqueous solution. Our results are consistent with our other published manuscripts, e.g., examining the formation stability constant [VO­(mIDA)­(dmbipy)] (mIDA = *N*-methyliminodiacetate anion, dmbipy = 4,4′-dimethoxy-2,2′-bipyridine), whose log β = 16.04.^29^ Comparing the literature data with other oxovanadium­(IV) acetylacetonate compounds, the complex formation constant for [VO­(acac)­(oda)]^−^ is log β = 16.45, while for [VO­(acac)­(Hoda)], it is log β = 21.14 (oda = oxidiacetate anion).^30^

Further confirmation of complex formation is provided by conductivity studies. A slight decrease in electrical conductivity from 231 to 224 μS/cm was observed, indicating a reduced amount of ions in the aqueous solution and the formation of the oxovanadium­(IV) complex. As titrant is added, the electrical conductivity increases due to an increase in the concentration of OH^–^ ions in the solution.

(Co)­polymerization and oligomerization process

Complex 1 in combination with Et_2_AlCl and EtAlCl_2_ was used as a catalyst for ethylene polymerization and ethylene/1-octene copolymerization. All reactions were carried out under the same set of conditions, i.e., at 60 °C in hexane for 10 min. Moreover, ethyl trichloroacetate (ETA), which enabled to overcome the problem of quick deactivation of vanadium catalysts by reactivation of inactive vanadium­(II) centers to active vanadium­(III) species,^2^ was added at ETA/V (molar ratio) = 200. The results are listed in (Table 3).^2^ Primary trials were conducted with 1/Et_2_AlCl (Table 3, entries D-1–3) to evaluate the effect of the Al/V molar ratio. When the ratio was increased from 1000 to 3000, the catalyst activity increased gradually, reaching the highest value equal to 83,400 kg mol_V_^–1^ h^–1^. Therefore, further investigations were conducted at an Al/V ratio of 3000. Thermal and structural characterizations of the obtained products, performed using DSC and FT-IR methods (Figure S7), indicate the formation of typical high-density polyethylene. The catalyst turned out to be very sensitive to the presence of a comonomer (entries D-4–6). Its activity decreased apparently with the increase of 1-octene concentration, showing a negative “comonomer effect”.^31^ At the same time, the comonomer incorporation into the polymer chains increased from 1.79 to 4.96%_mol_ for 1 and 5 mL of 1-octene in the feed, respectively, whereas the melting points and crystallinity of the copolymers decreased (Table 3). Increasing the monomers concentration ratio by decreasing the ethylene pressure increased comonomer incorporation in the produced copolymer as well (entry D-9). The exemplary DSC thermograms of produced copolymers are shown in Figure 3A. Narrow melting peaks indicate high compositional homogeneity (narrow comonomer distribution) of copolymers produced with 1/Et_2_AlCl.^32^ The microstructure of copolymer chains was determined for the copolymer with the highest comonomer incorporation (sample D-4) with ^13^C NMR spectroscopy. As can be seen in Figure 3B, only signals assigned to the isolated 1-octene units (14.03, 22.87, 27.34, 30.00, 30.50, 32.20, 34.62, and 38.26 ppm) are present. Therefore, the copolymer contains the EOE, OEE, OEEE, EOEE, and EEE sequences.^33^

**Table 3:** Results of Ethylene Polymerization and Ethylene/1-Octene Copolymerization Carried out with Complex **1** ^f^

entry	ethylene pressure, bar	*V*, μmol	activator, mmol		Al/V	1-octene, mL	yield, g	activity^a^	*T*_m_, °C^b^	*C*, %^b^	[1-octene], %_mol_ ^c^	*n*CH_3_/1000CH_2_^d^
D-1	5	1	Et_2_AlCl	1.0	1000		3.35	20,100	135.1	66.4		2.07
D-2	5	0.5	Et_2_AlCl	1.0	2000		3.20	38,400	134.2	69.6		1.04
D-3	5	0.3	Et_2_AlCl	0.9	3000		4.17	83,400	135.2	66.0		1.03
D-4	5	0.3	Et_2_AlCl	0.9	3000	5	0.45	9000	111.7	36.5	4.96	
D-5	5	0.3	Et_2_AlCl	0.9	3000	2	1.16	23,200	122.6	47.0	2.54	
D-6	5	0.3	Et_2_AlCl	0.9	3000	1	1.79	35,800	123.8	47.5	1.79	
D-7	5	0.3	EtAlCl_2_	0.9	3000		2.26	45,200	137.4	58.3		0.28
D-8	5	0.3	EtAlCl_2_	0.9	3000	2	0.83	16,600	112.0	26.2	^e^	
D-9	3	0.3	Et_2_AlCl	0.9	3000	2	1.21	24,200	116.5	42.3	4.46	

a Polymerization activity, kg·mol_V_^–1^·h^–1^.

b Melting point and crystallinity determined by the DSC method.^26^

c 1-Octene incorporation determined by FTIR.^27^

d Number of methyl groups per 1000 methylene groups in the polymer determined by FTIR.

e Mixture of products (copolymer and 1-octene homopolymer).

f Polymerization conditions: hexane 100 mL, polymerization temperature 60 °C, reaction time 10 min, ETA/V molar ratio = 200.

**Figure 3:**
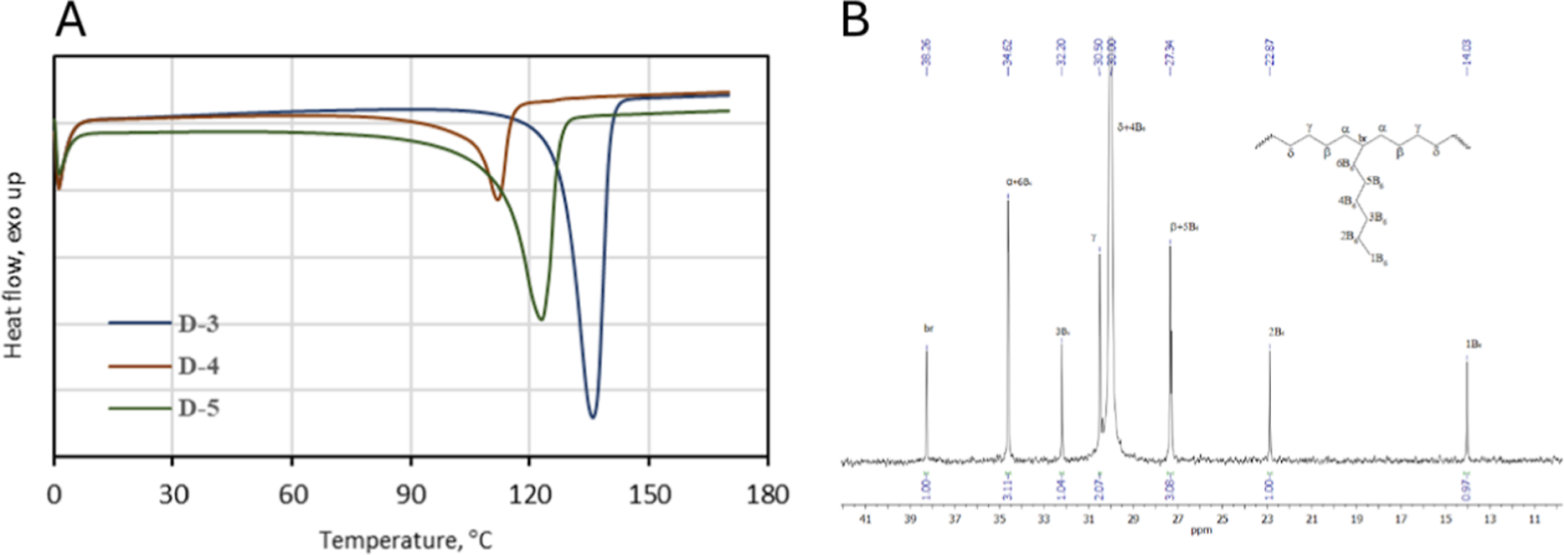
DSC thermograms of PE (D-3) and copolymers (D-5 and D-4) synthesized with **1**/Et_2_AlCl (a). ^13^C NMR spectrum of the ethylene/1-octene copolymer produced by **1**/Et_2_AlCl (sample D-4) (b).

Changing the activator from Et_2_AlCl to EtAlCl_2_ (entries D-7 and D-8) leads to a reduction in the activity of the catalytic system in both homo- and copolymerization. Moreover, analytical results (Figure S8) suggest that a mixture of products was formed in copolymerization, as was observed earlier for vanadium catalysts activated with dichloroethylaluminum.^34^ Polymeric materials obtained by­(co)­polymerization of ethylene or ethylene/1-octene in the presence of Et_2_AlCl are characterized by regular shapes—nanoplatelets (27 nm thick) with a smooth surface and spherical agglomerates with sizes of 2–7 μm (Figure S9).The synthesized PE and PE/1-octene were found to be highly thermally stable up to 400 °C (Figures S10 and S11).

To select suitable conditions to carry out the oligomerization process, the variable parameters were temperature (30/60/90 °C) and the molar ratio of Al/V (1000/2000/3000/4000) (Table S2). Using methylaluminoxane (MAO) as an activator and the polar monomers, i.e., 2-propen-1-ol, 2,3-dibromo-2-propen-1-ol and 3-buten-2-ol, the catalytic activity was higher than using trimethylaluminum (TMA) as cocatalyst. The exception was the oligomerization reaction of 2-chloro-2-propen-1-ol with TMA as an activator, where more than four times higher catalytic activity was obtained than when using methylaluminoxane as a cocatalyst (Table 4). As expected, much lower catalytic activities were obtained in the oligomerization reaction of polar monomers than when olefins were used. It is possible to coordinate hydroxyl groups to the active centers of the catalyst and poison it, especially without protecting groups, i.e., silanes or triisobutylaluminum (TIBA).

**Table 4:** Results of Polar Monomers Oligomerization Carried out with Complex [VO­(acac)_2_(3-ppy)]

entry	activator	nonomer	*T*_m_^a^ (°C)	*T*_g_^a^ (°C)	catalytic activity (kg mol^–1^ h^–1^)
D-10	MAO	2-propen-1-ol	109.19		1060
D-11		2,3-dibromo-2-propen-1-ol	71.48; 194.50		1840
D-12		2-chloro-2-propen-1-ol	128.71		340
D-13		3-buten-2-ol	115.07		260
D-14	TMA	2-propen-1-ol	103.09	–83.21	420
D-15		2,3-dibromo-2-propen-1-ol	102.07	–103.66	1560
D-16		2-chloro-2-propen-1-ol	133.10		1380
D-17		3-buten-2-ol	117.23		280

a Determined by DSC; Al/V = 3000; *T* = 60 °C; *t* = 30 min.

The morphology of the polymeric materials obtained is shown in SEM images (Figure S9). All oligomers obtained using MAO and TMA as cocatalysts show a typical discontinuous structure and are characterized by a rough surface.^35,36^ Using 2-propen-1-ol, 2-chloro-2-propen-1-ol, and 3-butene-2-ol, irregular crystalline structures (about a few μm in size) were obtained regardless of the cocatalyst, which may suggest the amorphous–crystalline nature of the synthesized oligomers. In the case of the oligo­(2-chloro-2-propen-1-ol), there are noticeable spherical voids.^35^ In the case of oligomeric materials obtained by using TMA instead of MAO, a higher surface roughness can be observed. A significant increase in the roughness of polar oligomer samples was observed compared to polyethylene or ethylene-1-octene copolymers.

STEM–EDX analysis with elemental mapping, i.e., C, O [in the case of oligo­(2-propen-1-ol)] and C, O, Cl [in the case of oligo­(2-chloro-2-propen-1-ol)] has been carried out (Figures 4 and S43–S45). By analyzing TEM images, the presence of carbon and oxygen can be confirmed; additionally, the presence of chlorine can be confirmed in the halogenated oligomer of allyl alcohol. The distributions of carbon and oxygen are homogeneous and uniform in both samples. Elemental composition and mass analyses were carried out by using an EDX detector. In the 3-buten-2-ol oligomer, there are 75.25% wt of carbon and 24.74% wt of oxygen (Table S3, Supporting Information). On the other hand, in the 2-chloro-2-propen-1-ol oligomer, lower mass values of carbon were observed than in oligo­(3-buten-2-ol), while the presence of 0.17 wt % % of chlorine is noteworthy. In the oligo­(2-chloro-2-propen-1-ol) sample, contamination with organoaluminum compounds was observed, probably originating from the cocatalyst. It is likely that the presence of polar atoms can lead to interaction with chemical individuals in the reaction mixture and side reactions with the cocatalyst, among others, resulting in the formation of small amounts of organoaluminum compounds in the polymeric material.

**Figure 4:**
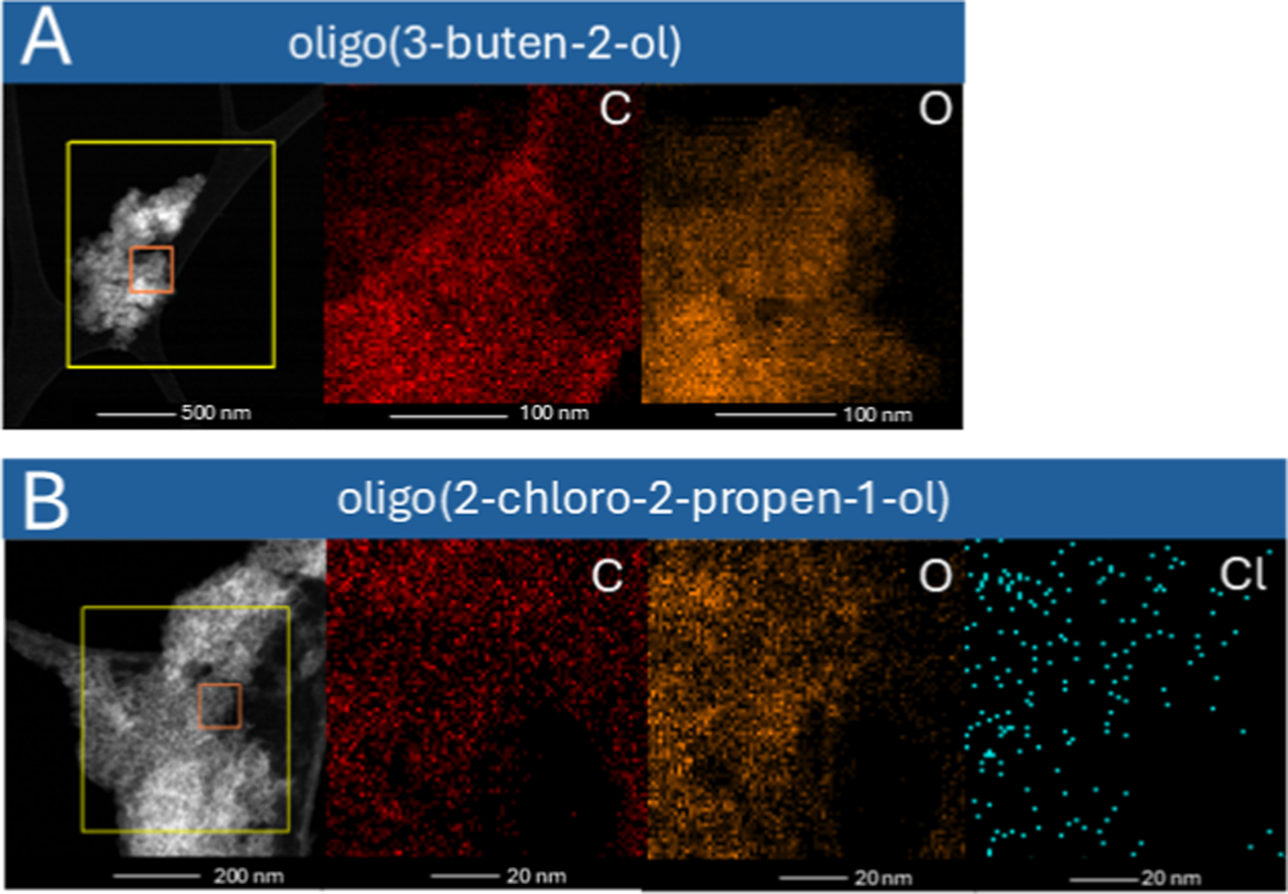
STEM images with EDX mapping area highlighted in orange for oligo­(3-buten-2-ol) (a) and oligo­(2-chloro-2-propen-1-ol) (b).

Analyzing the MALDI-TOF-MS spectra (Figure S12–S19), it is possible to determine how many mers polar oligomers consist of. The highest number of mers (4–17) was in the oligomerization reaction of 2-propen-1-ol catalyzed by 1/MAO. In contrast, the fewest were in the oligomerization reaction of 2,3-dibromo-2-propen-1-ol catalyzed by 1/TMA (2–4 mers). Analyzing FT-IR spectra, characteristic bands originating from functional groups in the synthesized polymeric materials can be observed, e.g., ∼3500 cm^–1^ (O–H), ∼3000 cm^–1^ (C–H), ∼1500 cm^–1^ (–CH_2_), and ∼1100 cm^–1^ (C–O) (Figures S20–S25). On the other hand, the change from plastic to crystalline form was only observed for oligomeric materials synthesized using 1/TMA. The *T*_g_ values are −83.21 °C and −103.66 °C for 2-propen1-ol and 2,3-dibromo-2-propen-1-ol oligomers, respectively (Figures S26–S41). Polar oligomers are characterized by wider, often bimodal melting points compared to polyethylene. Determinant factors may be the high degree of dispersity, the presence of crystallites, the different degree of crystallinity probably less crystalline than polyethylene due to its lower melting temperature, and the heterogeneous composition of the oligomers.^37,38^

### 3.3 N_2_ and CO_2_ Capture—Sorption Properties of Obtained (Co)­polymers and Oligomers

The isotherms of the oligomers 2-propen-1-ol and 2-chloro-2-propen-1-ol obtained in the presence of **1**/MAO or **1**/TMA and 3-buten-2-ol in the presence of **1**/TMA have a similar shape with adsorption–desorption hysteresis at total nitrogen capacities of 7.2, 7.7, 8.2, 0.4, and 1.7 mmol g^–1^, respectively (Figure 5 and Table 5). At low *p*/*p*_0_ values, the nitrogen volume increases rapidly. It then adopts a more linear form, which suggests multilayer adsorption and is typical of type-IV isotherms. In contrast, the isotherm for oligomers of 2,3-dibromo-2-propen-1-ol synthesized in the presence of **1**/MAO and **1**/TMA shows a type III isotherm. This indicates a gradual increase in the adsorbate volume with increasing pressure values, up to a maximum of 1.5 and 0.3 mmol g^–1^ respectively. This analysis suggests weak interactions between the adsorbent (N_2_) and the oligomeric material (Table 5).^39^ The BET surface area of the synthesized HDPE is similar to literature data (41.85 m^2^ g^–1^).^40^

**Figure 5:**
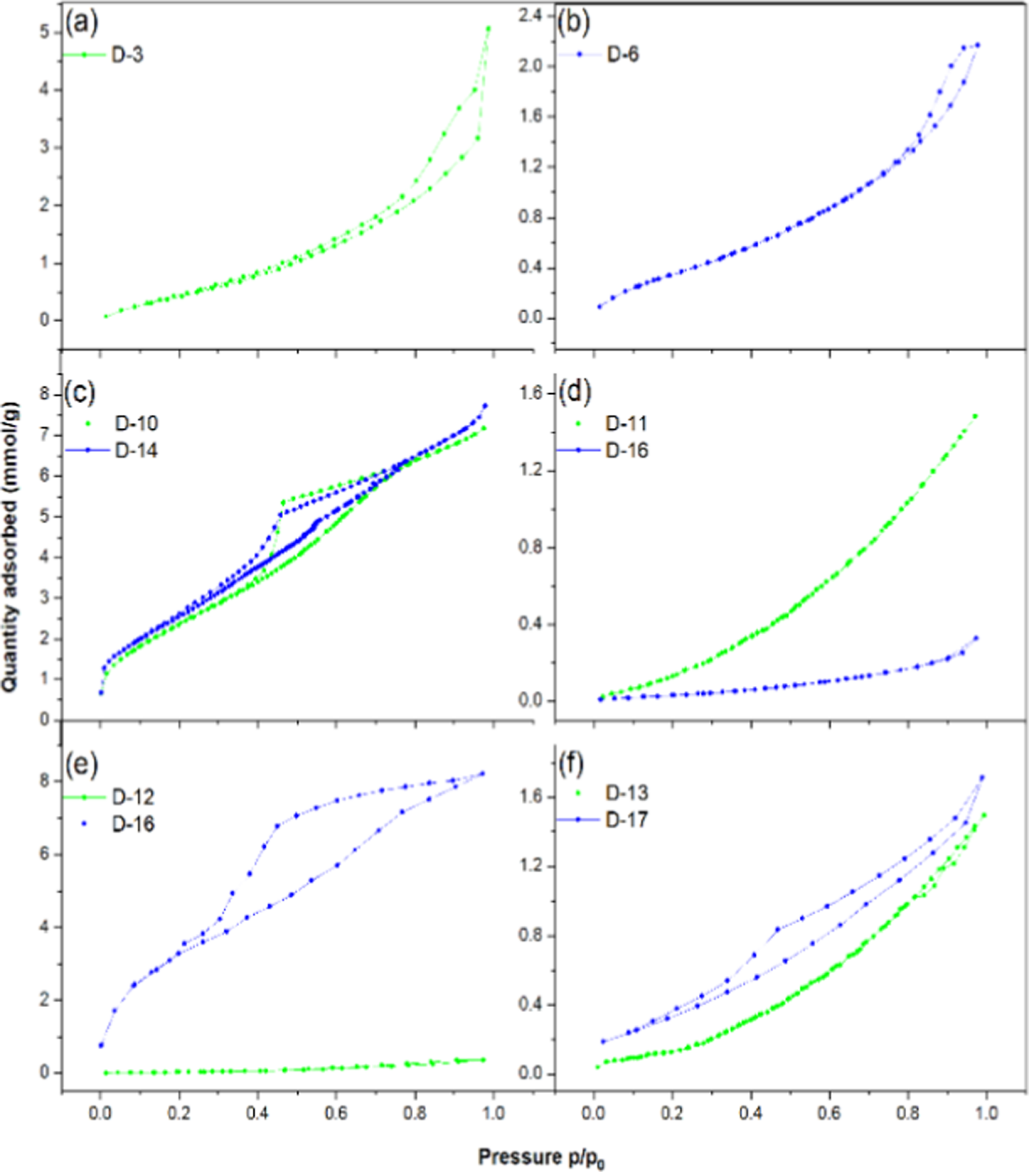
Nitrogen adsorption and desorption isotherms of polymeric materials: (a) polyethylene (D-3), (b) ethylene/1-octene copolymer (D-6), (c) 2-propen-1-ol oligomer synthesized using 1/MAO (D-11) and 1/TMA (D-14), (d) 2,3-dibromo-2-propen-1-ol oligomer synthesized using 1/MAO (D-11) and 1/TMA (D-16), (e) 2-chloro-2-propen-1-ol oligomer synthesized using 1/MAO (D-12) and 1/TMA (D-16), and (f) 3-buten-2-ol oligomer synthesized using 1/MAO (D-13), 1/TMA (D-17).

**Table 5:** Specific Surface Area, Pore Size, and Carbon Dioxide Sorption of Synthesized Polymeric Materials (Materials Degassed in 60 °C)

entry	activator	(co)polymer/oligomer	BET surface area (m^2^ g^–1^)	pore volume at *p*/*p*_0_ ∼ 1 (cm^3^·g^–1^)	the most common pore width (nm)	CO_2_ sorption at *p*/*p*_0_ ∼ 1 bar in 298 K (mmol g^–1^)
D-3	AlEt_2_Cl	polyethylene	58	0.18	3.1	0.38
D-6		polyethylene/1-octene	37	0.08	2.9	0.10
D-10	MAO	2-propen-1-ol oligomer	205	0.25	2.4	0.78
D-11		2,3-dibromo-2-propen-1-ol oligomer	35	0.07	3.1	0.14
D-12		2-chloro-2-propen-1-ol oligomer	5	0.01	2.6	0.07
D-13		3-buten-2-ol oligomer	12	0.05	4.4	0.82
D-14	TMA	2-propen-1-ol oligomer	224	0.27	5.8	0.76
D-15		2,3-dibromo-2-propen-1-ol oligomer	5	0.01	3.0	0.07
D-16		2-chloro-2-propen-1-ol oligomer	269	0.28	2.1	0.38
D-17		3-buten-2-ol oligomer	34	0.59	2.7	0.55

The synthesized (co)­polymers and oligomers were tested for CO_2_ sorption properties (Figure S46). Degassing of polymeric materials (preheated) was carried out at 60 °C (Table 5). Due to previously performed DSC and TGA analyses, we observed that oligomers are not very stable at high temperatures (for example, Figure S32 thermal decomposition of 2-propen-1-ol oligomer around 70 °C), so to prevent thermal degradation we prepared samples at relatively low temperatures. The highest CO_2_ sorption (at ∼1 bar at 298 K) was observed for the oligomers 3-buten-2-ol in the presence of **1**/TMA (0.82 mmol g^–1^ CO_2_), **1**/MAO (0.47 mmol g^–1^ CO_2_) and 2-propen-1-ol in the presence of **1**/MAO (0.78 mmol g^–1^ CO_2_) and **1**/TMA (0.76 mmol g^–1^ CO_2_). The reason for the observed high sorption (compared to other materials) in the oligomers of 3-buten-2-ol (0.82 mmol g^–1^) and 2-propen-1-ol (0.76 mmol g^–1^) is probably the most common pore width, the presence of only hydroxyl groups (without additional chlorine and bromine atoms), and the purity of the sample, as confirmed by TEM–EDX analysis. For oligo­(3-buten-2-ol) the most common pore width is 4.4 nm and for oligo­(2-propen-1-ol) 5.8 nm. In addition, Yaghi et al. suggested that materials with weak nucleophilic functional groups could serve as sorbents for the reversible formation of hydrogen carbonate.^41,42^ The authors described the synthesis of CD-MOF-2 consisting of the renewable cyclic oligosaccharide γ-cyclodextrin and RbOH or MAF-X25 and MAF-X27ox. In contrast, carbon­(IV) oxide sorption for both materials is at a similar level.

Research on the best sample, oligo­(3-buten-2-ol) was extended to other sorption parameters, i.e., heat of adsorption and CO_2_/N_2_ selectivity. The sample with the highest sorption properties to carbon­(IV) oxide and the sorption of N_2_ and CO_2_ was measured at 25 °C. The CO_2_/N_2_ selectivity for oligo­(3-buten-2-ol) is 22.38 (Figure 6B). The recycling of oligo­(3-buten-2-ol) was carried out in 5 cycles. The CO_2_ sorption of the polymeric material decreases slightly and after five cycles it decreases by about 20% (Figure 6A). Perhaps despite the annealing between cycles, the pores are clogged with carbon­(IV) oxide. For the 3-buten-2-ol oligomer obtained using TMA, additional sorption studies were carried out at temperatures of 273 and 223 K. From the isotherms, the heat of adsorption of CO_2_ by the polymer was determined, which was equal to 8.51 kJ mol^–1^ (in the CO_2_ adsorption range of 7–16 cm^3^ g^–1^)^43^ which can be attributed to physisorption (Figure 6C).^44,45^

**Figure 6:**
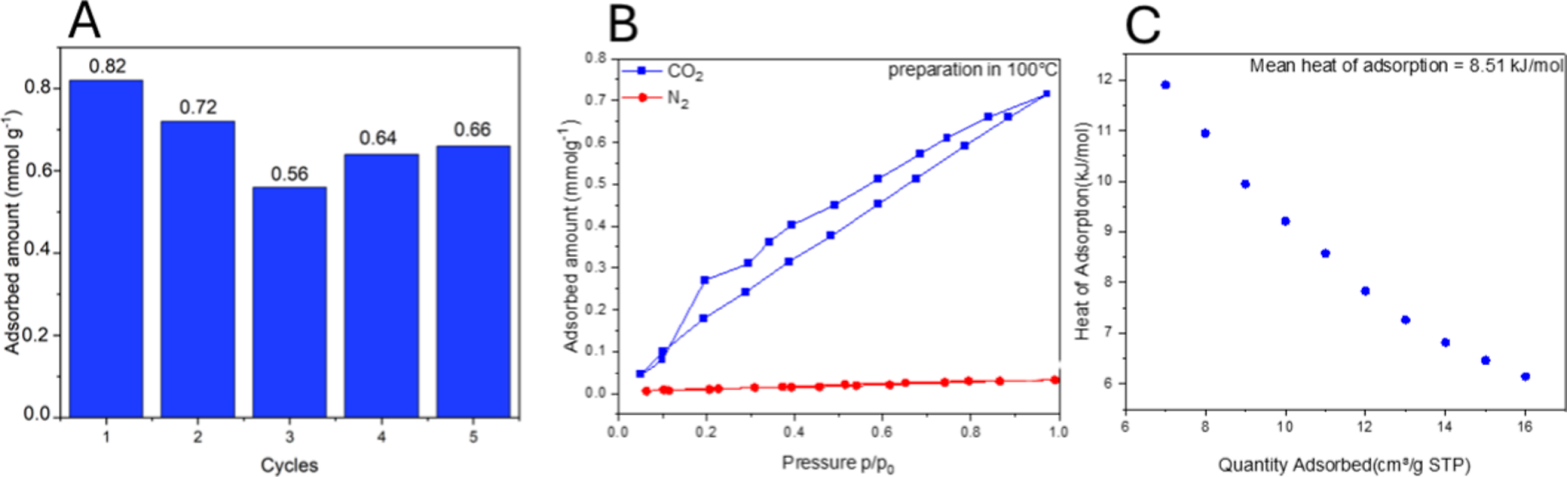
Stability of oligo­(3-buten-2-ol) at 5 cycles (a), CO_2_/N_2_ selectivity at 298 K of oligo­(3-buten-2-ol) (b), and effect of the quantity adsorbed on the isosteric heat of adsorption of carbon­(IV) oxide onto oligo­(3-buten-2-ol) (c).

On the other hand, oligo­(3-buten-2-ol) stored for 6 months after reheating at 100 °C exuded increased BET surface area and much higher CO_2_ sorption (4.34 mmol g^–1^). The specific surface area of BET increased from 12 m^2^ g^–1^ to 98 m^2^ g^–1^ (Figure S47, Supporting Information). We decided to use the quenched sample as a carbon­(IV) oxide sorbent for several cycles. After the fourth cycle, we observed a 62% decrease in CO_2_ sorption (Figure S48, Supporting Information). As a result, the polymer material is not very stable. The reason for the increase in the CO_2_ sorption properties with increased annealing temperature may be the removal of toluene from the pores of polymeric materials. Toluene was used as a reaction medium in the oligomerization reaction of polar monomers and could contribute to pore clogging. Due to the structure and the presence of the aromatic ring, it is difficult to remove from low thermally stable oligomers. Therefore, polymerization studies using hexane as a solvent are preferred.

## 4 Discussion

Comparing the catalytic activity of compound **1** with that of other ligand-based complexes β-diketones for example [Zr­(Cp)­(acac)_3_] (Cp = cyclopentadienyl) showed catalytic activity (7100 kg_PE_ mol_Zr_^–1^ h^–1^).^46^ Using 5 atm of ethylene and a temperature of 70 °C, polyethylene with low dispersity (*M*_w_/*M*_n_ = 2.48) was synthesized. The authors indicate that the higher catalytic activity and a higher degree of alkylation using MAO is influenced by the larger distance between Zr–O (for [Zr­(Cp)­(acac)_3_] equals 2.26 Å compared to the analogues of the Zr­(IV) complex the distance between atoms is 1.95 Å).^46^ Other examples are nickel­(II) and cobalt­(II/III) catalytic materials based on β-diketones, i.e., Co­(R_1_COCHCOR_2_), which have also been successfully used in ethylene dimerization reactions.^47^ It is proven that activity decreases according to the relation R_1_, R_2_ = Ph, Ph > CH_3_, CH_3_ > Ph, CH_3_ > CF_3_, CF_3_ using TMA as an activator. In contrast, using a catalytic system based on Ni­(acac)_2_, Et_2_AlCl and PPh_3_ (molar ratio 1:7:3) at 25 °C, 95.4 wt % conversions were obtained by the ethylene dimerization reaction. Noteworthy is the high selectivity of obtaining 2-butenes (93.8%).^47^ Comparing the catalytic activity of compound **1** in the ethylene polymerization reaction with other complexes based on the vanadium­(IV) ion, it was observed that it is one of the highest catalytic activities reported to date. To the best of our knowledge, high thermal stability is responsible for the high catalytic activity, so that the olefin polymerization process can be carried out at high temperatures and high chemical stability of complex compound **1**. Compared with VO­(acac)_2_, compound *cis*/*trans*-[VO­(acac)_2_(3-ppy)] synthesized by our group is 678 times more active in the ethylene polymerization reaction. This indicates that the bond between the free electron pair on the nitrogen atom of 3-ppy and the VO­(IV) ion affects the stabilization of the active site of the catalyst and increases the physicochemical stability by which the catalytic system is subsequently deactivated. In addition, the presence of 3-phenylpyridine as an electron-donor ligand probably contributed to increased reactivity and spatial hindrance, so that the active center of the catalyst was not so quickly poisoned. Additional confirmation of the above statement is the correlation of the results with the thermodynamic stability of the oxovanadium­(IV) complex (log β_1210_ = 27.82). This confirms that complex **1** is highly thermodynamically stable, which has the effect of limiting the deactivation of the active center of the catalyst and changing the oxidation step from IV to II (inactive). Excessively high stability is not always desirable due to the limitation of reactivity and reaction kinetics and hindering the generation of active centers after alkylation by the organoaluminum compound. Comparing the catalytic activity of **1** with other V­(IV) ion-based postmetallocene precatalysts, it was observed that the coordination number is also of great importance. The use of five-coordination iminovanadium­(IV) complexes with phosphine ligands by Lorber and Leone et al.^48^ contributed to the synthesis of highly active precatalysts (Figure 7). Nevertheless, the resulting catalytic activity is more than 38.4 times lower than that of complex *cis*/*trans*-[VO­(acac)_2_(3-ppy)]. The enhanced catalytic activity of the six-coordinated complexes over the five-coordinated compounds is shown by studies using dipicolinate oxovanadium­(IV) compounds with *N*-donor ligands, i.e., 1,10-phenanthroline and 2,2̀-bipyridyl.^49^ This is also an additional confirmation that the vanadium­(IV) ion forms a more stable bond with oxygen (V=O) than with nitrogen (V=N). The reason is probably the higher electronegativity of oxygen than nitrogen. Unfortunately, the rigidity of precatalyst *cis*/*trans*-[VO­(acac)_2_(3-ppy)] also affected the low incorporation of the comonomer into the polyolefin chain. A negative effect of comonomer was observed, by which, as the concentration of 1-octene increased, the catalytic activity of 1 significantly decreased.

**Figure 7:**
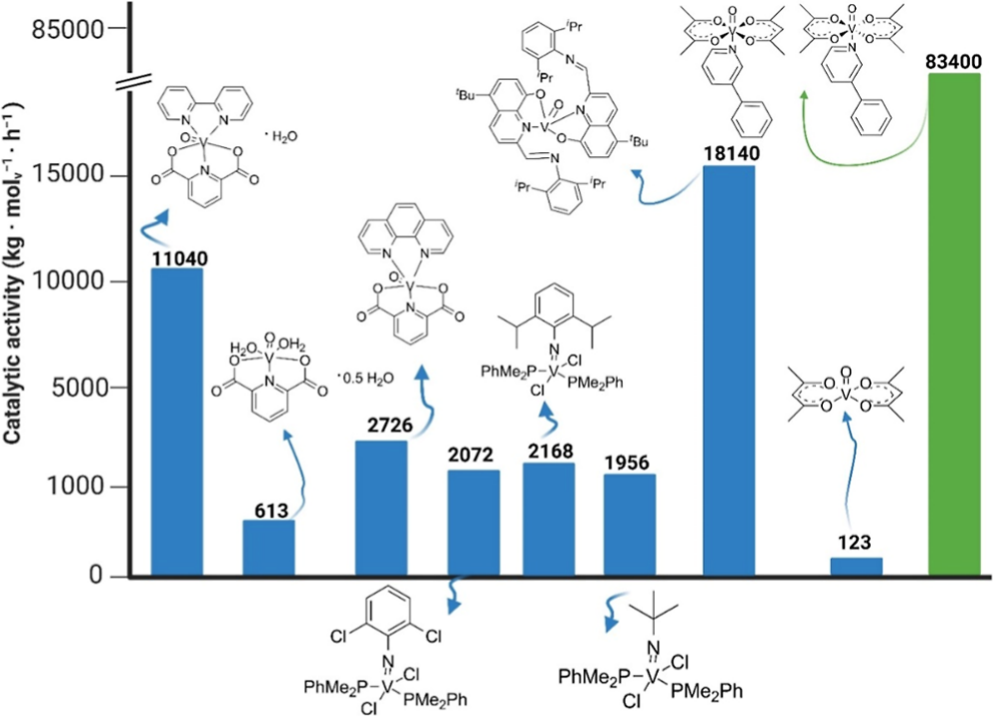
Catalytic activity of vanadium­(IV) coordination compounds in the ethylene polymerization reaction (blue—complexes known in the literature,^48–51^ green—this work). Created with BioRender.com.

## 5 Perspectives

The innovative compound **1** is highly active in olefin homopolymerization and oligomerization reactions of polar monomers. On the other hand, due to the rigidity of the catalytic system and the high spatial hindrance, efficient incorporation of comonomers into the olefin chain was hindered. Future research should focus on designing a suitable catalytic system that provides both high catalytic activity in homo- and copolymerization reactions of olefins. In particular, the functionalization of polyolefins with polar monomers seems to be a promising direction due to the possibility of 3D printing, the application of such macromolecules as nanoporous membranes, materials with high impact resistance, or pH sensors.^52^ The synthesized oligomers represent a new generation of gas sorption materials, including CO_2_ and N_2_. It is worth leaning toward further investigation of their environmental applications including SO_2_ sorption, pH sensors, or membranes. Polymers containing heteroatoms (C–O, C–N), such as PET and polyurethanes, have lower activation energy and are easier to recycle than polyolefins (PE, PP), which have very low recoverability (<10%) due to permanent C–C and C–H bonds.^53^ Therefore, oligomers containing hydroxyl groups, i.e., oligo­(2-propen-1-ol) and oligo­(3-buten-2-ol), should be amenable to chemical recycling, show low environmental impact, and should be more biodegradable than polyolefins. On the other hand, modeling on known polymers containing chlorine atoms, i.e., PVC oligomers such as oligo­(2-chloro-2-propen-1-ol) or bromine derivatives of oligo­(allyl alcohol), may be problematic due to toxic combustion products (i.e., HCl, Cl_2_, dioxins). The direction of further research should oscillate around the study of the life cycle and emission calculus of the production of synthesized macromolecular compounds.

## 6 Conclusions

For the first time, an innovative coordination compound, characterized by *cis* and *trans* geometric isomerism, which ensures high physicochemical stability in the oligo- and polymerization reaction of olefins, was synthesized. The presence of an auxiliary ligand (3-phenylpyridine) in the molecular structure of *cis*/*trans*-[VO­(acac)_2_(3-ppy)] increased the reactivity of the coordination compound and steric hindrance, which correlates with higher catalytic activity than in previously reported works.^50^ This is confirmed by the very high activity in the ethylene polymerization reaction (83,400 kg mol_V_^–1^ h^–1^) ethylene/1-octene copolymerization (35,800 kg mol_V_^–1^ h^–1^) and in the oligomerization reaction of polar monomers like allyl alcohol (1060 kg mol_V_^–1^ h^–1^). Analyzing the literature data, it can be concluded that the obtained value of a catalytic activity is from 7.6 to 1390 times higher than that of postmetallocene precatalysts reported so far based on the vanadium­(IV) cation in the ethylene polymerization reaction (Figure 7 and Table S4, Supporting Information). *Cis-*/*trans*-[VO­(acac)_2_(3-ppy)] is 678 times more active in the ethylene polymerization reaction than VO­(acac)_2_.^50^ Compound 1 is up to 3.8 times more active than oxovanadium­(IV) precatalysts with diglycolate anion and 1,10-phenanthroline as ligands in the oligomerization reaction of 2-chloro-2-propen-1-ol. The high catalytic activity can be linked to the high thermodynamic stability value of the oxovanadium­(IV) complex log β_1210_ = 27.82. This is a rare phenomenon because, despite the rapid poisoning of the active sites by the polar groups of monomers, the catalytic activity is still much higher than that of postmetallocene precatalysts described in the literature.^54^ In addition, a new generation of polymeric CO_2_ sorbents has been proposed. The synthesized oligomers of 2-propen-1-ol and 3-buten-2-ol exhibit the highest CO_2_ sorption properties (up to 0.82 mmol g^–1^). The heat of adsorption of CO_2_ by the oligo­(3-buten-2-ol) was equal to 8.51 kJ mol^–1^. It is expected that a breakthrough in polymer synthesis technology may, in the long run, influence the development of the sector of “clean” production of polymer materials with high sorption properties of toxic gases, such as CO_2_, N_2_O, or SO_2_.

## Supplementary Material



## Notes

All DSC and TGA curves, MALDI-TOF, FTIR, UV–vis spectra, TEM and SEM images, and analyses related to sorption properties (PDF)

## Abbreviations

3-ppy: 3-phenylpyridine
acac: acetylacetone
PE: polyethylene
Cp: cyclopentadienyl
DRS: ultraviolet–visible diffuse reflectance spectroscopy (UV–Vis DRS)
