# Supplementary material for: CO2 Capture by Porous Polymeric Sorbents: From New cis‑/trans-Oxovanadium(IV) Catalyst to Functional Materials
Source: J Phys Chem B. 2025 Jun 13;129(25):6253–64. doi: 10.1021/acs.jpcb.5c01060 (PMC12207570; doi:10.1021/acs.jpcb.5c01060)
Supplement: Supplementary file 1 [file jp5c01060_si_001.pdf]

Supporting Information for Publication

CO_2 Capture By Porous Polymeric Sorbents: From New *cis/trans*-Oxovanadium(IV) Catalyst To Functional Materials

Kacper Pobłocki[*a], Marzena Białek[b], Katarzyna N. Jarzembska[c], Mateusz A. Baluk[a], Radosław Kamiński[c], Przemysław Rybiński[d], Krzysztof Matus[e], Joanna Drzeżdżon[a], Barbara Gawdzik[d], Dagmara Jacewicz*[*a]*

[a]Department of Environmental Technology, Faculty of Chemistry, University of Gdansk Wita Stwosza 63, 80-308 Gdansk, Poland

[b] Department of Chemical Technology and Polymer Chemistry, Institute of Chemistry, Institute of Chemistry, University of Opole, Oleska 48, 45-052 Opole, Poland

[c] Department of Chemistry, University of Warsaw, Żwirki i Wigury 101, 02-089 Warsaw, Poland

[d]Institute of Chemistry, Jan Kochanowski University, Uniwersytecka 7, 25-406 Kielce, Poland

[e]Materials Research Laboratory, Silesian University of Technology, Konarskiego 18A, 44-100 Gliwice, Poland

Corresponding Authors

*E-mail address: (K.P): kacper.poblocki@phdstud.ug.edu.pl; (D.J): dagmara.jacewicz@ug.edu.pl

Table S1. Selected X-ray data collection, processing and refinement parameters for the crystal structure of [VO(acac)$_2$(3-phenylpyridine)].

Data set	**1**
Moiety formula	$C_{21}H_{23}NO_5V$
Moiety formula mass, M_r / a.u.	420.4
Crystal system	triclinic
Space group	$P\bar{1}$ (no. 2)
Z	4
F_{000}	876
Crystal colour & shape	small colourless block
Crystal size / mm^3	0.06×0.03×0.03
T / K	100
a / Å	7.870(2)
b / Å	14.069(3)
c / Å	18.965(4)
α / Å	83.56(3)
β / Å	79.37(3)
γ / Å	83.22(3)
V / Å^3	2040.6(8)
d_{calc} / g·cm^{-3}	1.368
θ range	2.38–76.14°
Absorption coefficient, μ / mm^{-1}	4.331
No. of reflections collected / unique	24431 / 8352
R_{int}	5.00%
No. of reflections with $I > 3\sigma(I)$	6065
No. of parameters / restraints / constraints	505 / 0 / 184
$R[F]$ ($I > 3\sigma(I)$)	3.99%
$R[F]$ (all data)	6.18%
$\varrho_{res}^{min/max}$ / e·Å^{-3}	−0.40 / +0.49
CCDC code	2316951

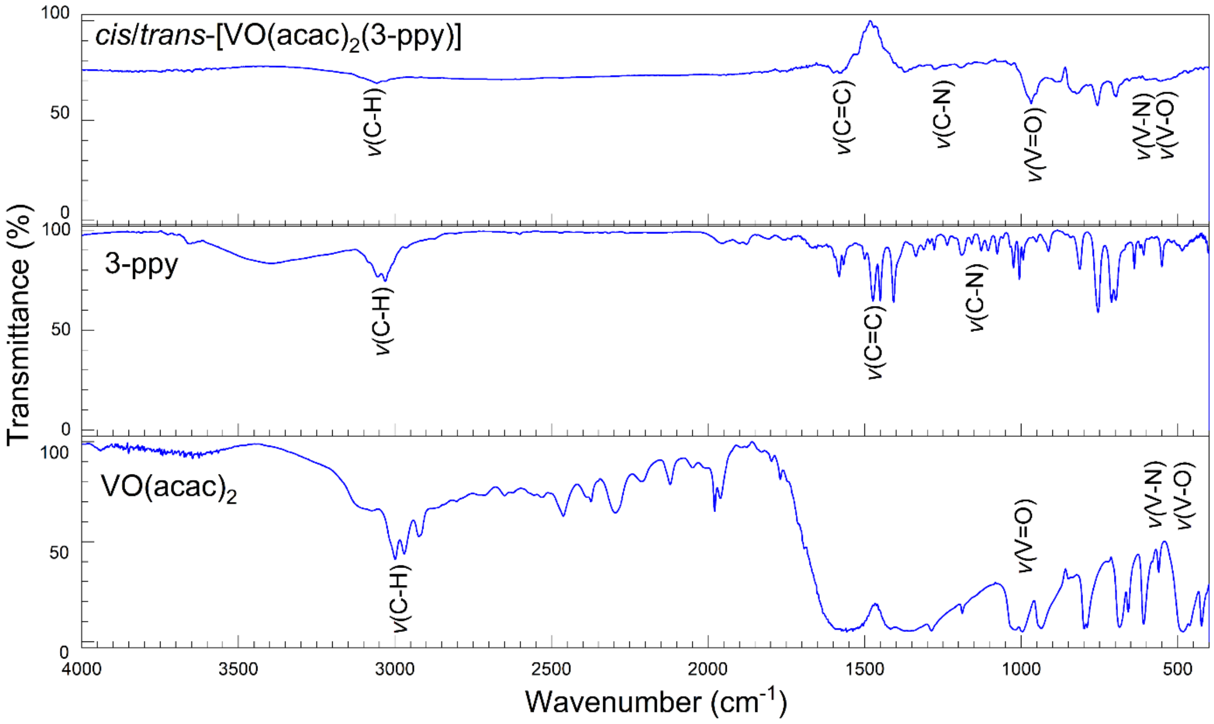

Figure S1. FT-IR spectrum of $VO(acac)_2$, 3-ppy and *cis*/*trans*-$[VO(acac)_2(3\text{-}ppy)]$.

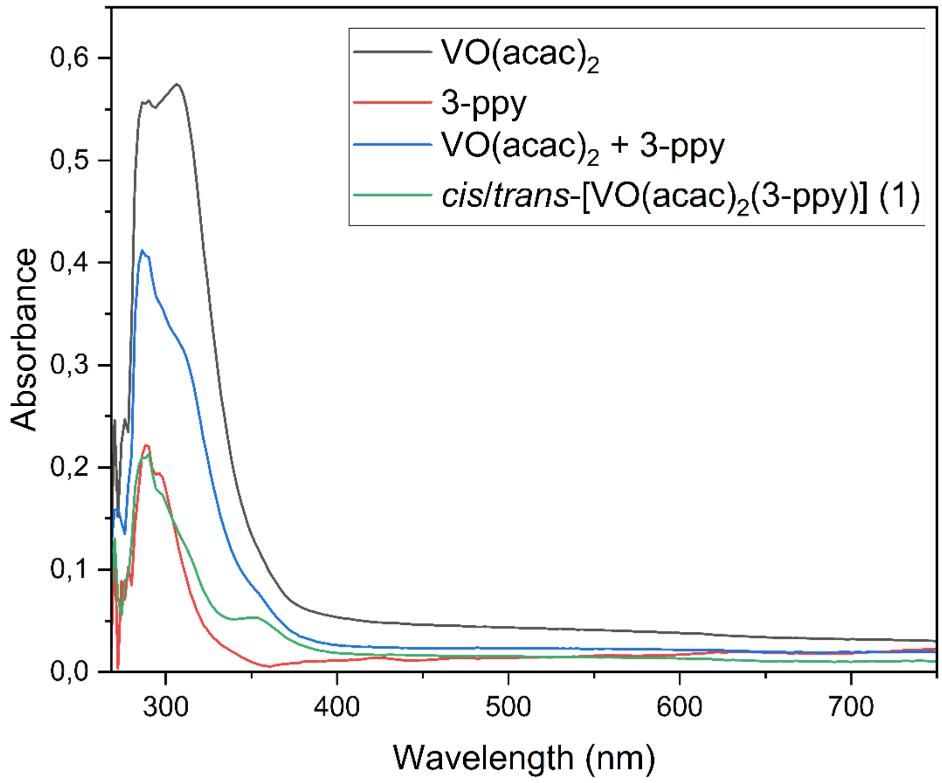

Figure S2. UV-VIS spectra of *cis/trans*-[VO(acac)$_2$(3-ppy)] (**1**), VO(acac)$_2$ and 3-ppy. All solutions were prepared in water: 0.1 mM VO(acac)$_2$, 0.1 mM 3-ppy, 0.05 mM *cis/trans*-[VO(acac)$_2$(3-ppy)].

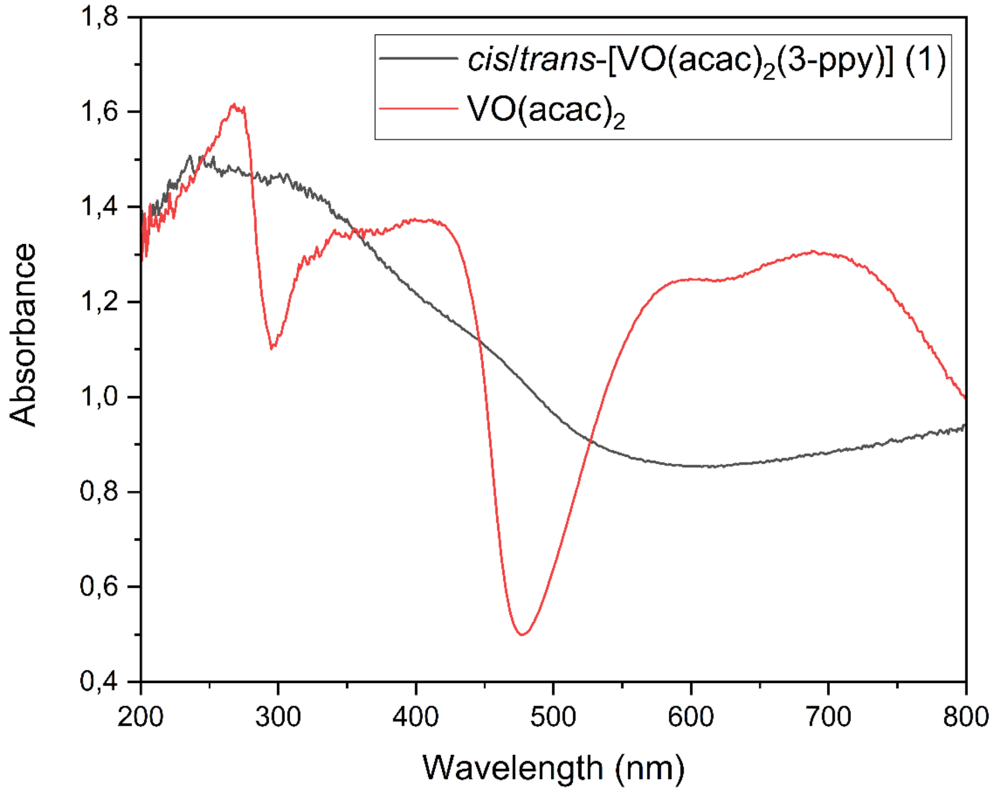

Figure S3. Ultraviolet–Visible Diffuse Reflectance Spectroscopy (UV–Vis DRS) of *cis/trans* [VO(acac)$_2$(3-ppy)] (**1**).

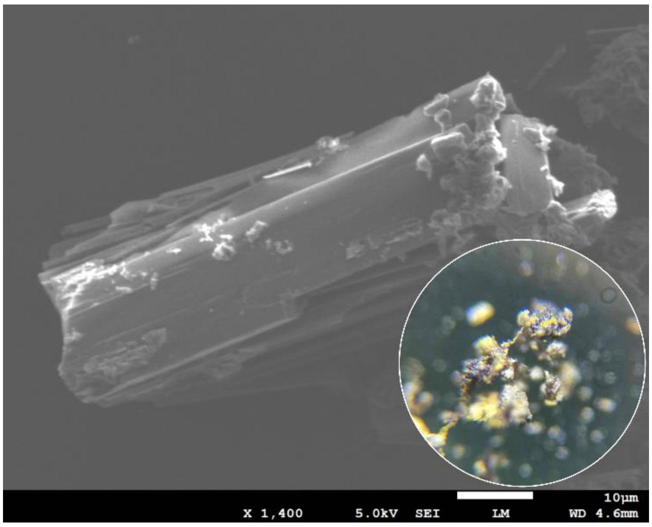

Figure S4. SEM images of *cis/trans*-[VO(acac)$_2$(3-phenylpyridine)] with a real photo using an optical microscope (x10) as insert.

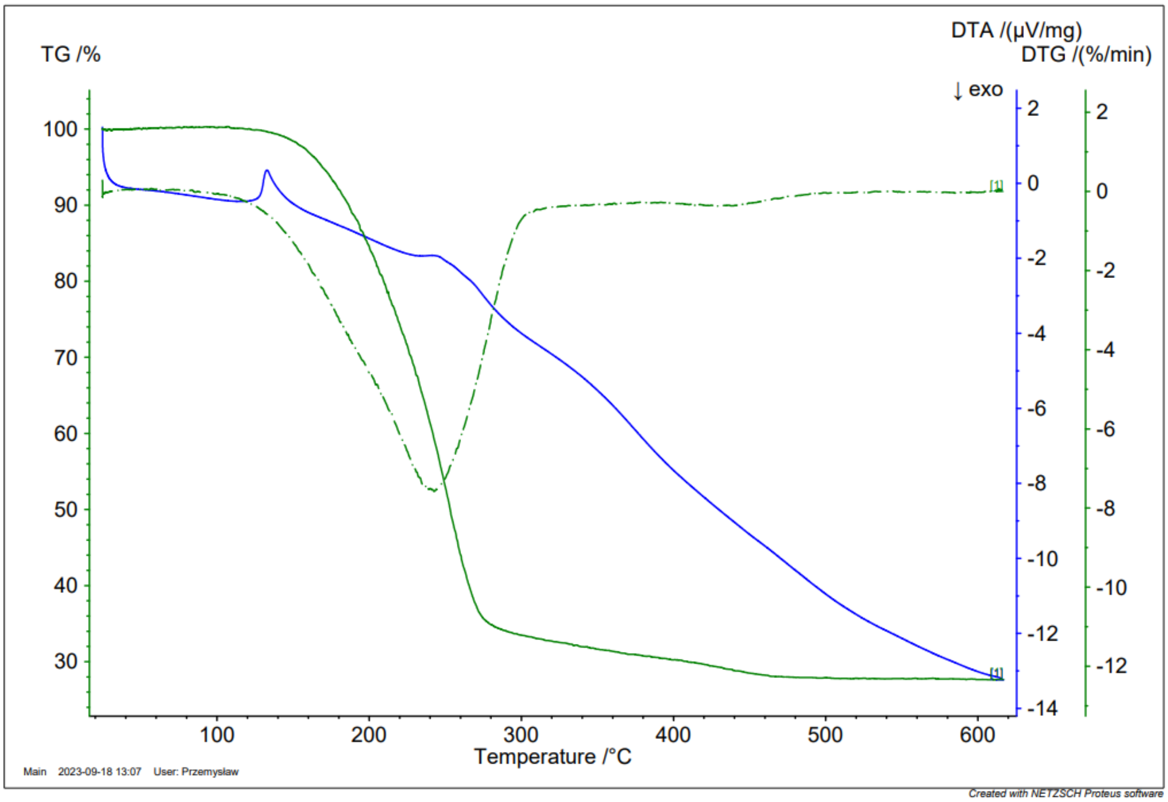

Figure S5. TGA curve of *cis*/*trans*-[VO(acac)$_2$(3-ppy)].

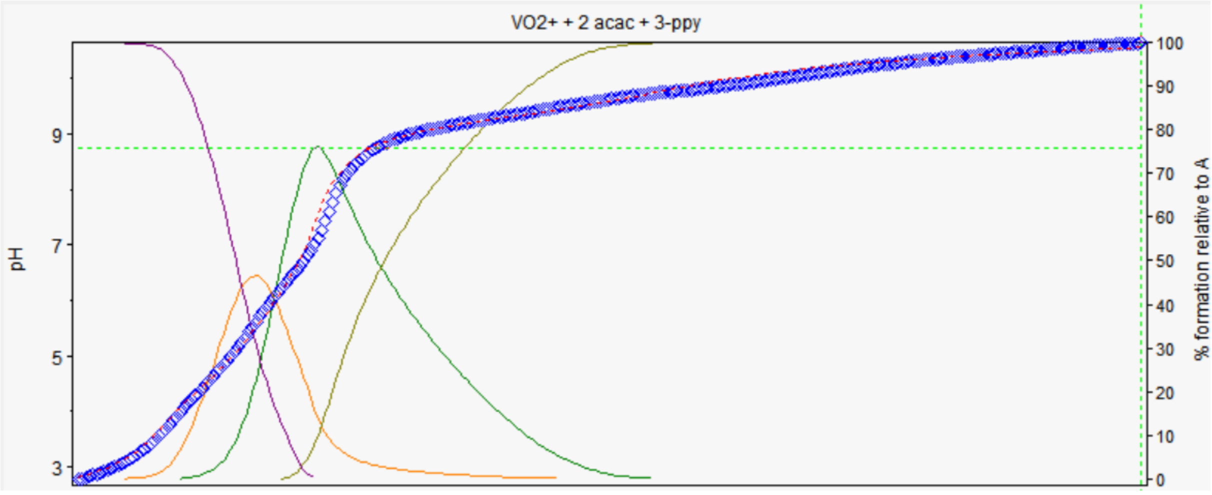

Figure S6. Potentiometric titration curve and fitting of the experimental to the theoretical curve in Hyperquad2008

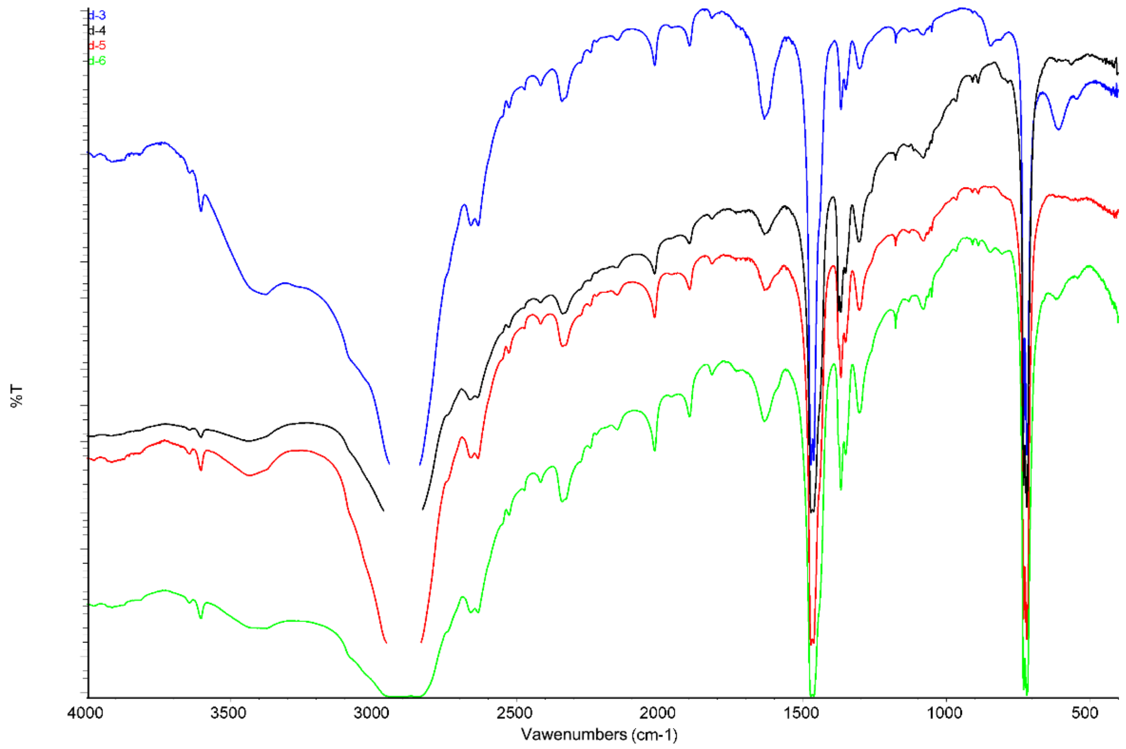
d-3
d-4
d-5
d-6
%T
4000
3500
3000
2500
2000
1500
1000
500
Vawenumbers (cm-1)

a)

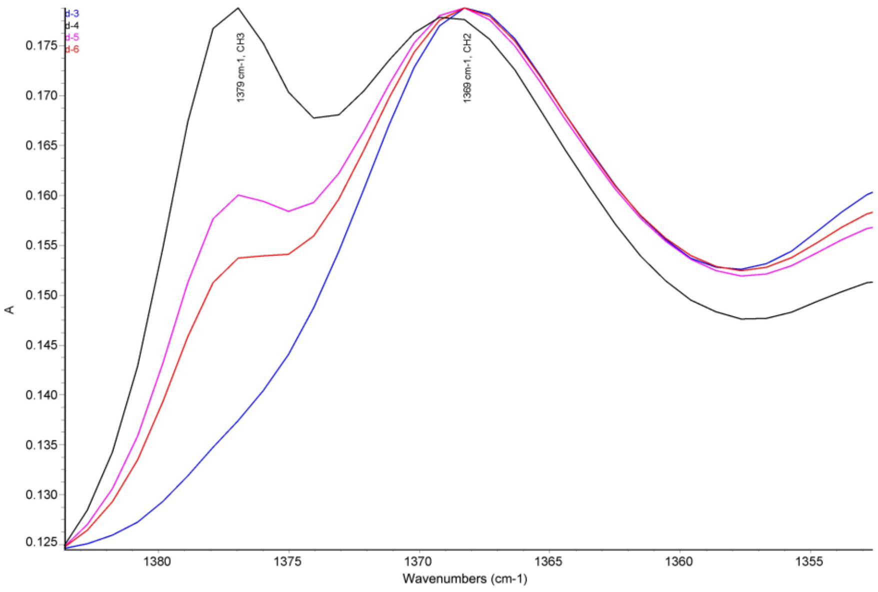

b)

Figure S7. The FTIR spectra of PE (sample D-3) and copolymers (sample D-4, D-5 and D-6) synthesized with complex [VO(acac)$_2$(3-phenylpyridine)]/Et$_2$AlCl: full spectrum (a) and zoom-in spectrum in the range of 1400-1300 cm^{-1}.

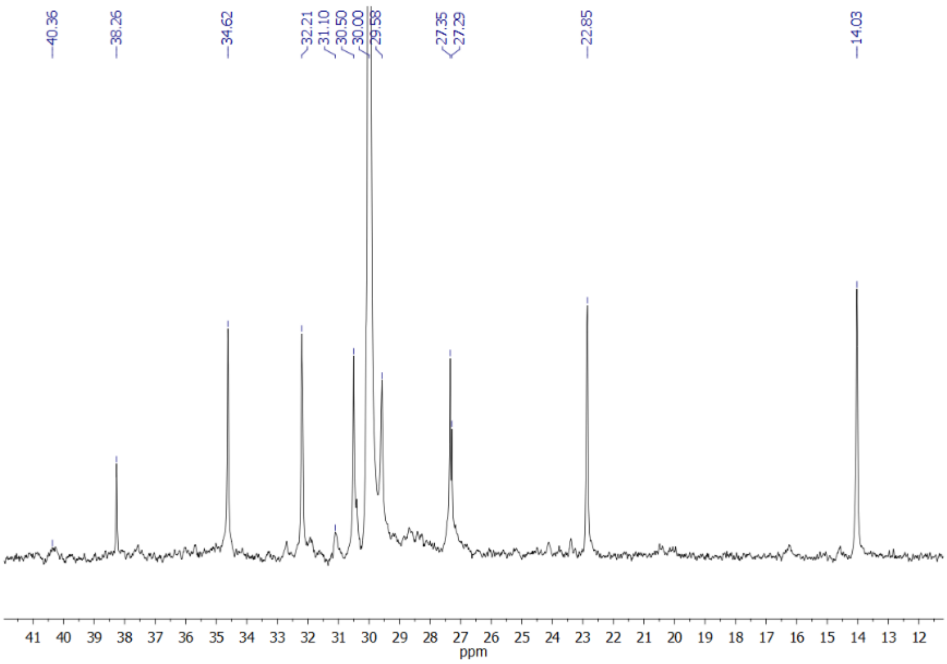

Figure S8. ^{13}C NMR spectrum of the product obtained in the ethylene/1-octene copolymerization process carried out in the presence of [VO(acac)$_2$(3-phenylpyridine)]/EtAlCl$_2$ (sample D-8).

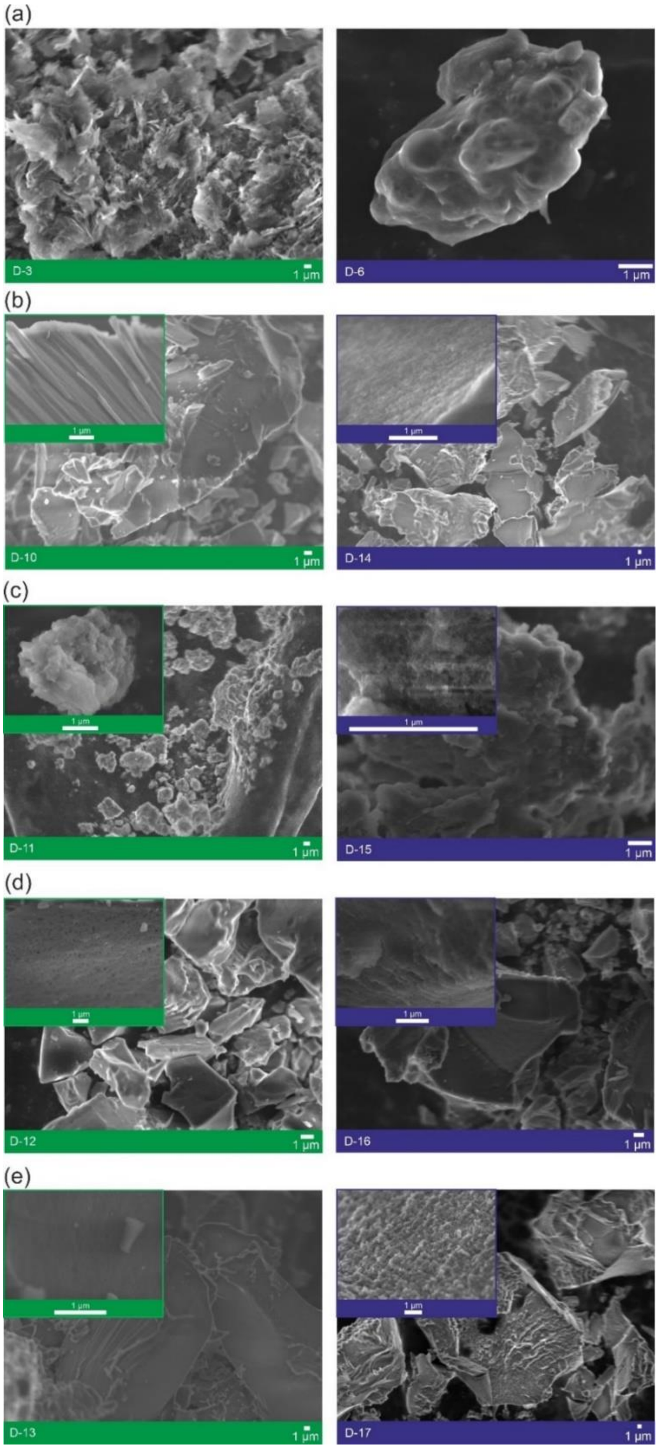

Figure S9. SEM images of synthesized polymer materials. (a) polyethylene synthesized using [VO(acac)$_2$(3-phenylpyridine)]/Et$_2$AlCl (D-3), (b) ethylene-1-octene copolymer synthesized using [VO(acac)$_2$(3-phenylpyridine)]/Et$_2$AlCl (D-6), (c) 2-propen-1-ol oligomer synthesized using using [VO(acac)$_2$(3-phenylpyridine)]/MAO (D-10) and [VO(acac)$_2$(3-phenylpyridine)]/TMA (D-14), (d) 2,3-dibromo-2-propen-1-ol oligomer synthesized using [VO(acac)$_2$(3-phenylpyridine)]/MAO (D-11) and [VO(acac)$_2$(3-phenylpyridine)]/TMA (D-15), (e) 2-chloro-2-propen-1-ol oligomer synthesized using [VO(acac)$_2$(3-phenylpyridine)]/MAO (D-12) and [VO(acac)$_2$(3-phenylpyridine)]/TMA (D-16), (f) 3-buten-2-ol oligomer synthesized using [VO(acac)$_2$(3-phenylpyridine)]/MAO (D-13), [VO(acac)$_2$(3-phenylpyridine)]/TMA (D-17).

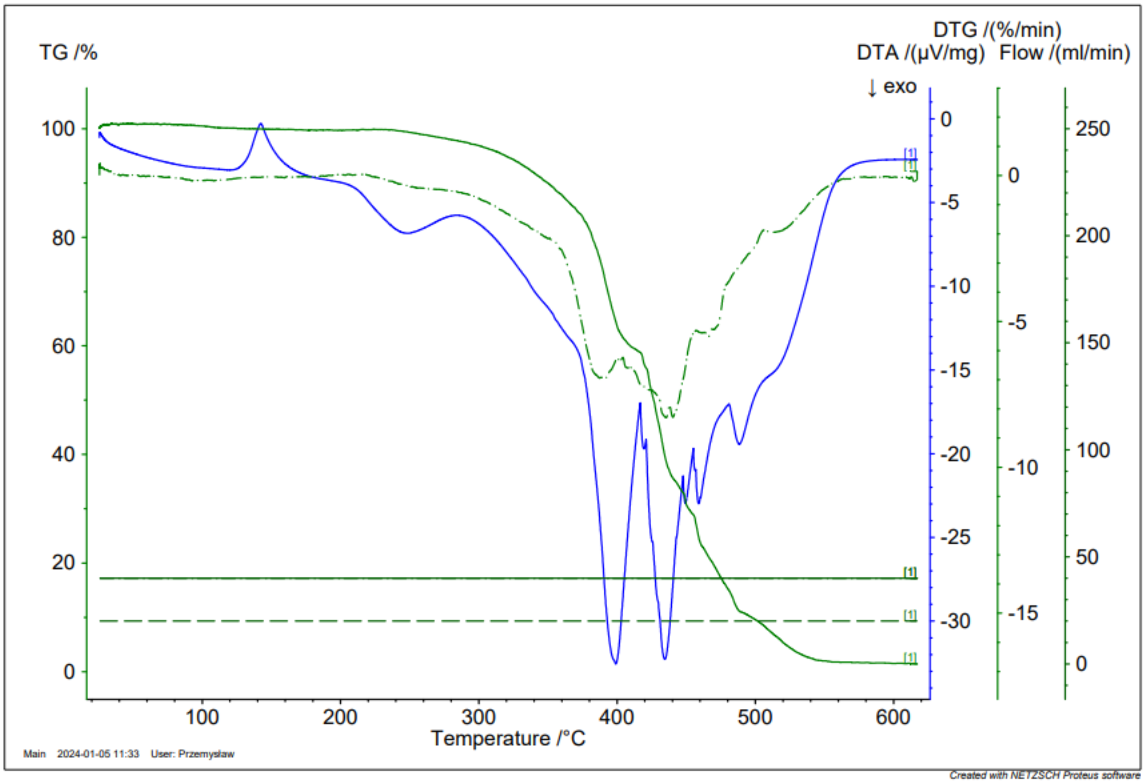

Figure S10. The TGA and DTA curves of polyethylene (D-3) obtained using [VO(acac)$_2$(3-phenylpyridine)]/Et_2AlCl as a catalyst.

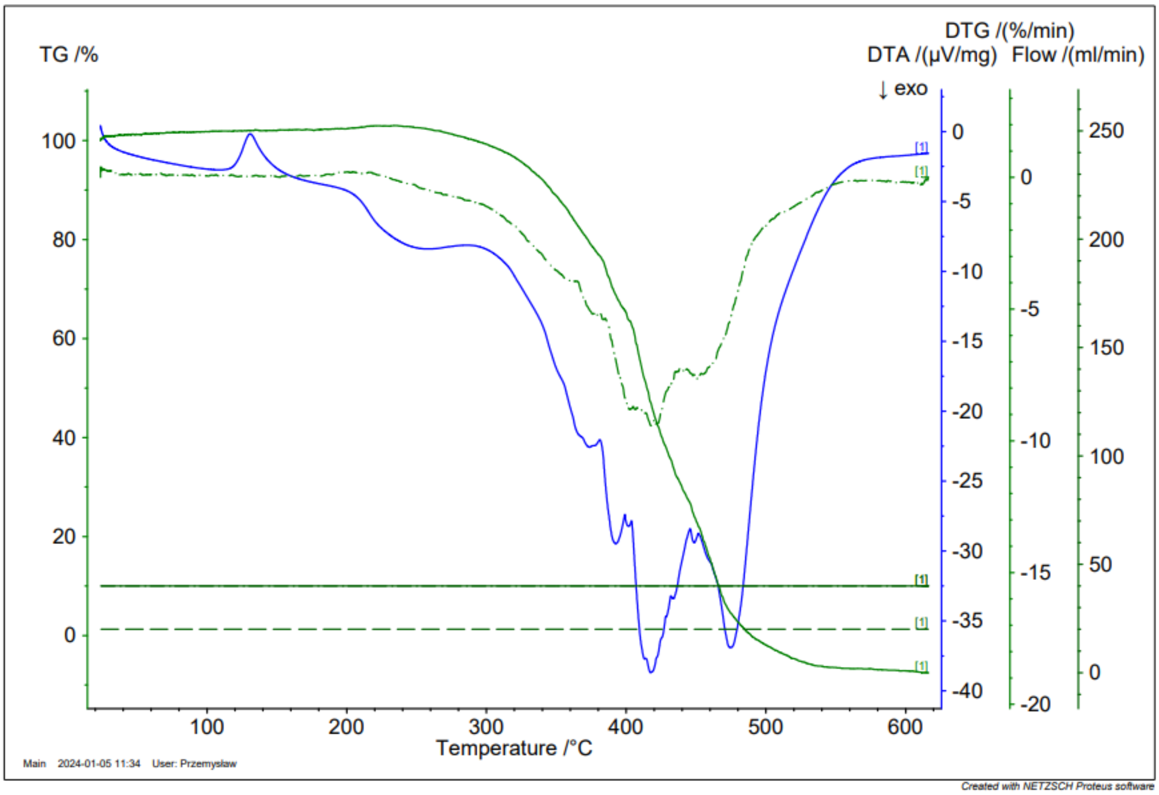

Figure S11. The TGA and DTA curves of copolymer ethylene/1-octene (D-6) using [VO(acac)$_2$(3-phenylpyridine)]/Et_2AlCl as a catalyst.

Table S2. Optimalization of oligomerization (2-propen-1-ol catalyzed by [VO(acac)$_2$(3-phenylpyridine)]/MAO).

**V (μmol)**	**Al/V**	**T (°C)**	**Yield (g)**	**Catalytic activity (kg mol^{-1} h^{-1})**
3	1:500	30	0.08	58.3
3	1:1000	30	0.23	153.3
3	1:1500	30	0.26	173.3
1	1:2000	30	0.17	340
1	1:2500	30	0.25	500
1	1:3000	30	0.3	600
1	1:3500	30	0.24	480
1	1:4000	30	0.25	500
1	1:3000	50	0.24	480
1	1:3000	60	0.53	1060
1	1:3000	90	0.4	800

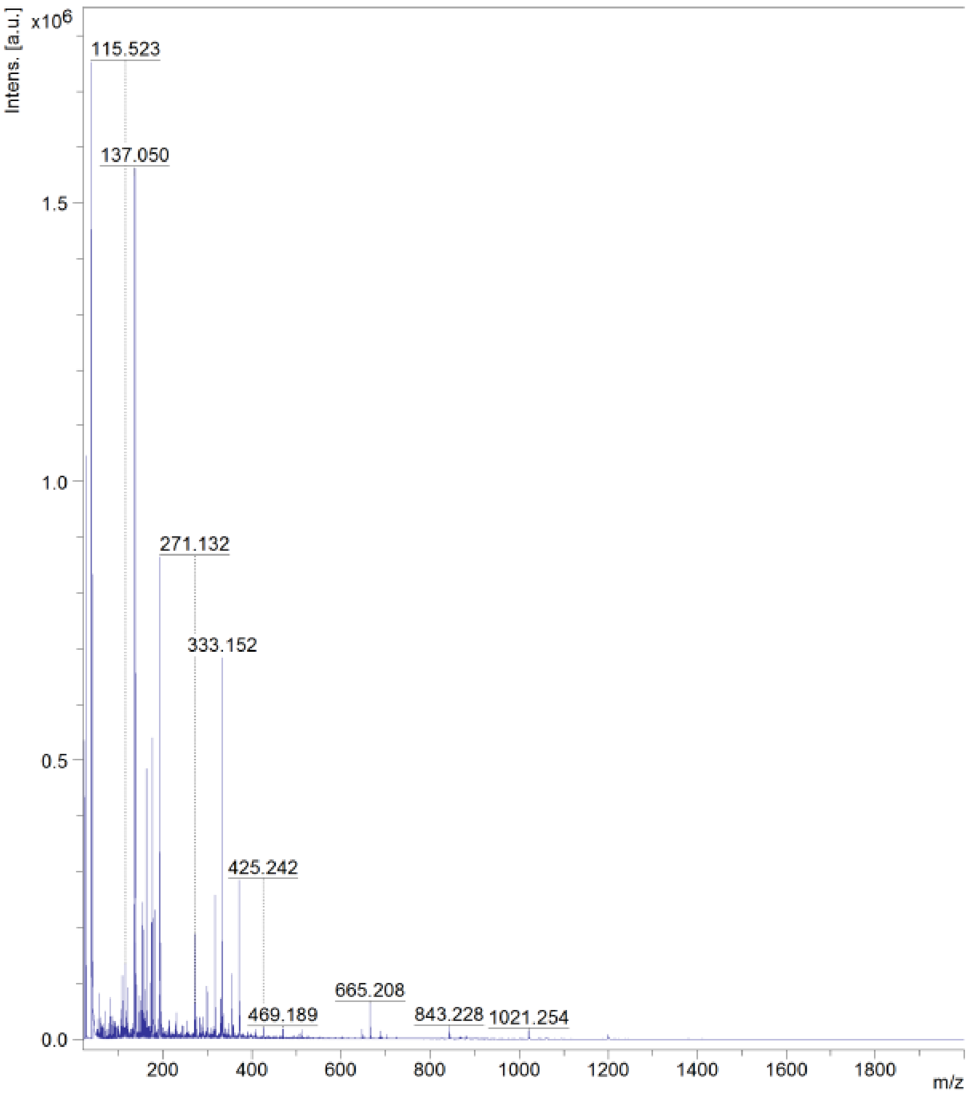

Figure S12. MALDI-TOF-MS spectrum of a 2-propen-1-ol oligomer synthesized using coordination compound [VO(acac)$_2$(3-phenylpyridine)] and MAO as activator (DHB matrix = 2,3-Dihydroxybenzoic acid).

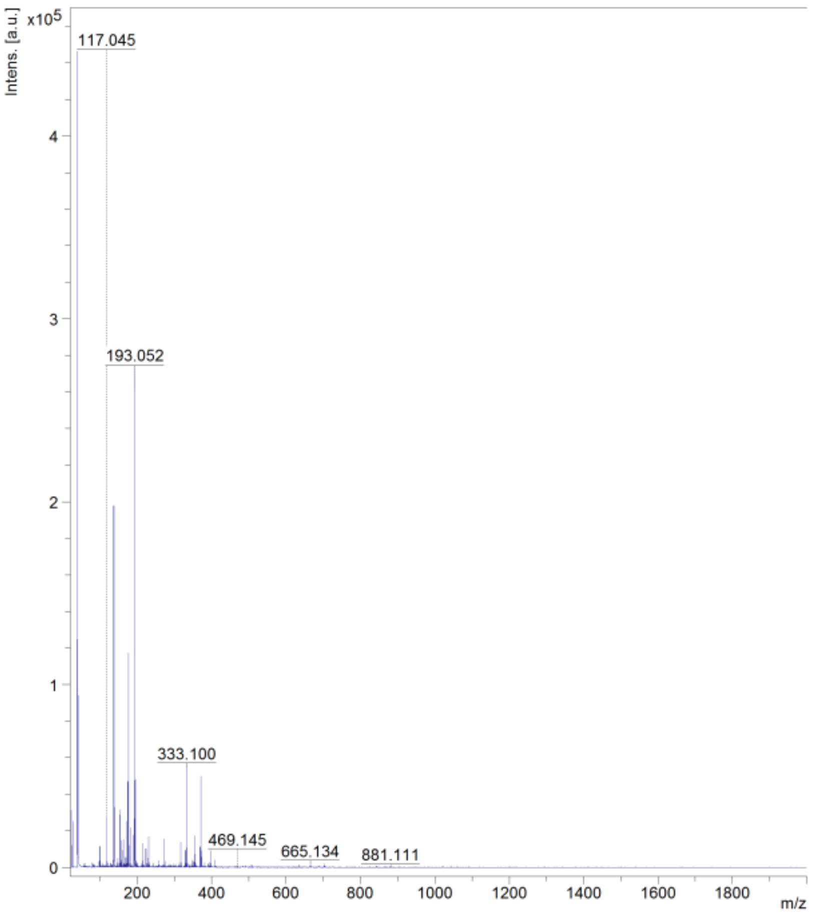

Figure S13. MALDI-TOF-MS spectrum of a 2-propen-1-ol oligomer synthesized using coordination compound [VO(acac)$_2$(3-phenylpyridine)] and TMA as an activator (DHB matrix).

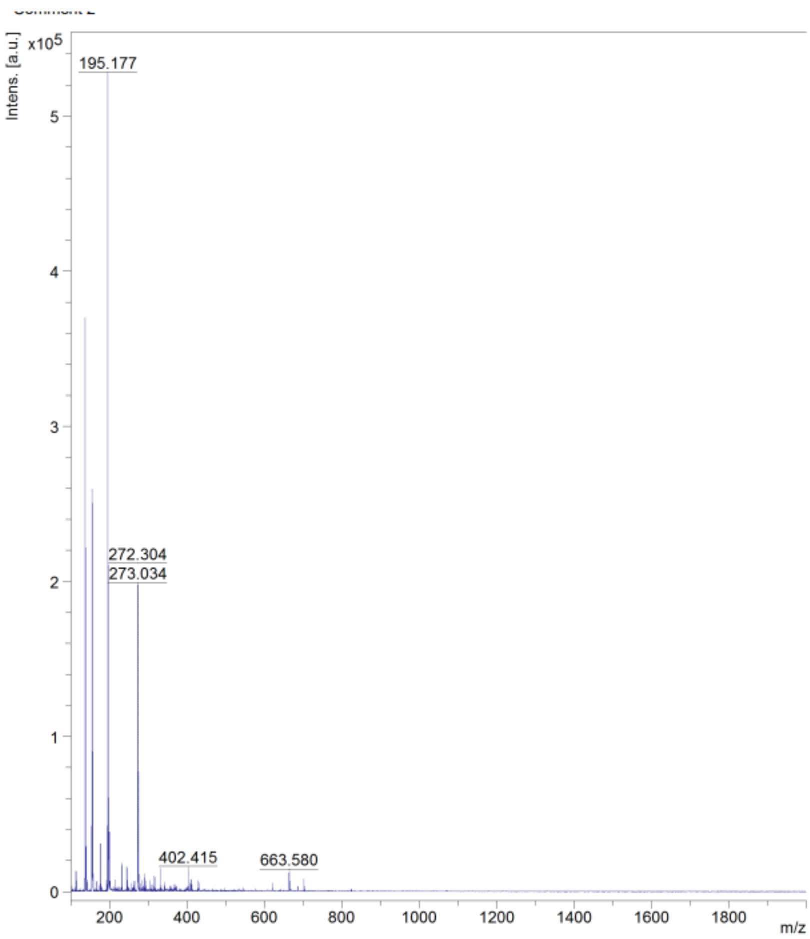

Figure S14. MALDI-TOF-MS spectrum of a 2-chloro-2-propen-ol oligomer synthesized using coordination compound [VO(acac)$_2$(3-phenylpyridine)] and TMA as an activator (DHB matrix).

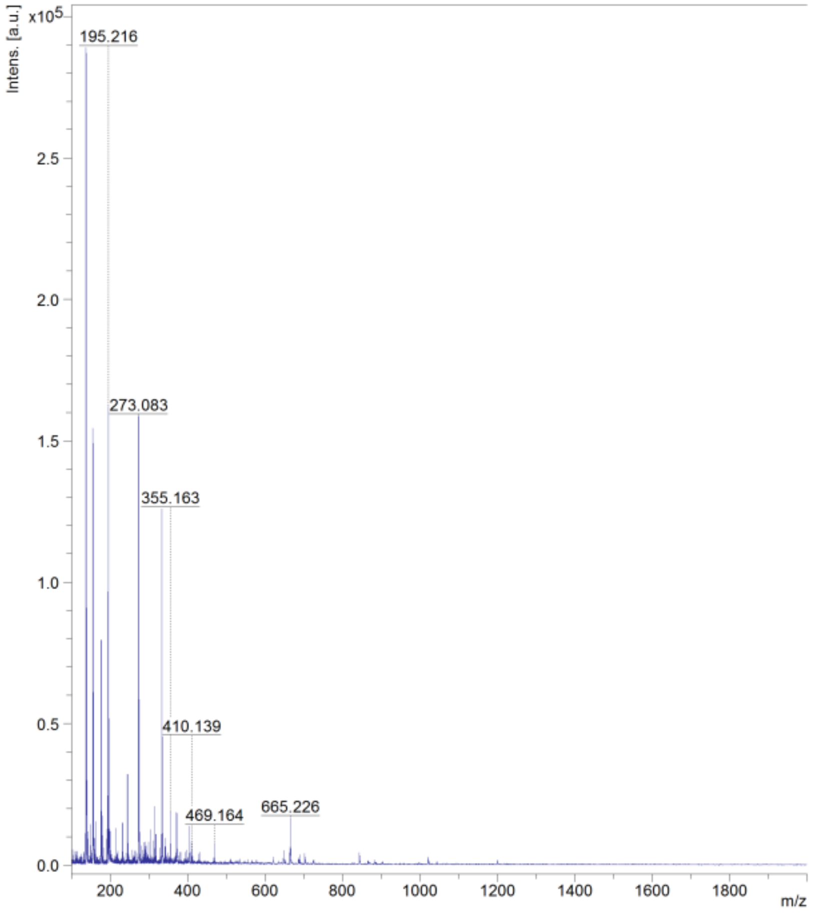

Figure S15. MALDI-TOF-MS spectrum of an oligomer of 2-chloro-2-propen-1-ol, synthesized using coordination compound [VO(acac)$_2$(3-phenylpyridine)] and MAO as an activator (DHB matrix).

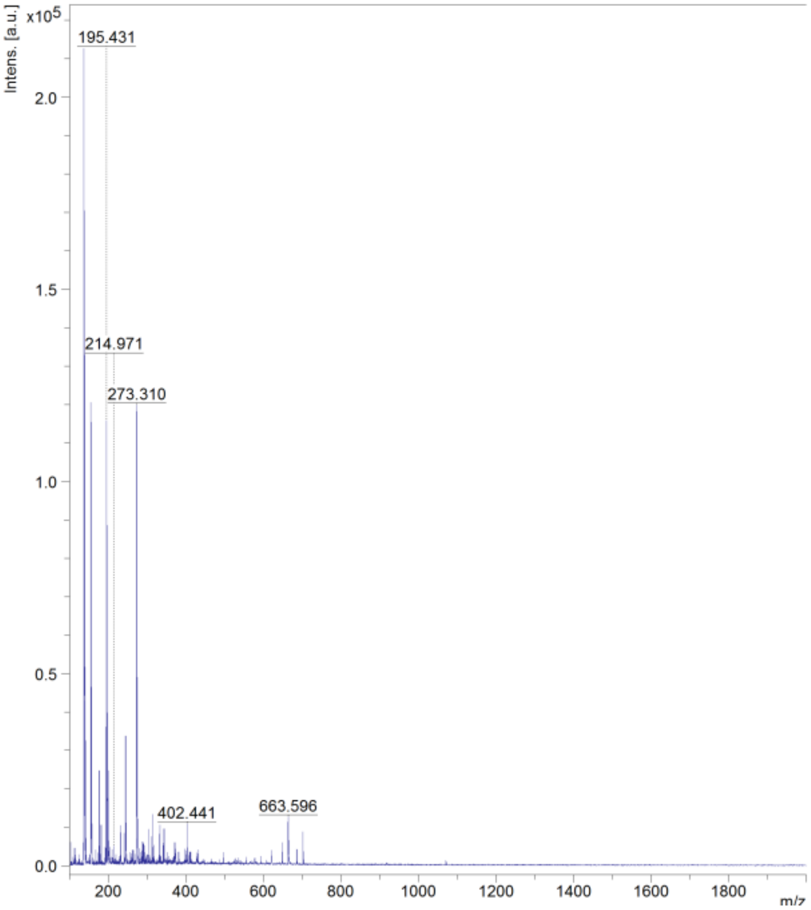

Figure S16. MALDI-TOF-MS spectrum of a 3-buten-2-ol oligomer synthesized using coordination compound [VO(acac)$_2$(3-phenylpyridine)] and TMA as an activator (DHB matrix).

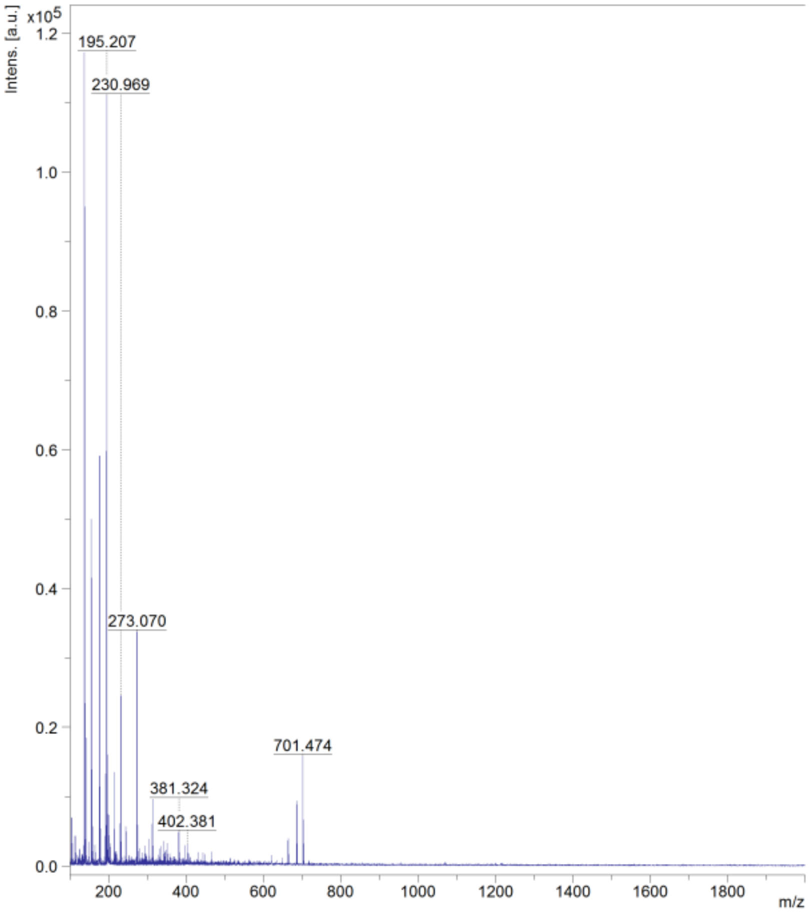

Figure S17. MALDI-TOF-MS spectrum of a 3-buten-2-ol oligomer synthesized using coordination compound [VO(acac)$_2$(3-phenylpyridine)] and MAO as an activator (DHB matrix).

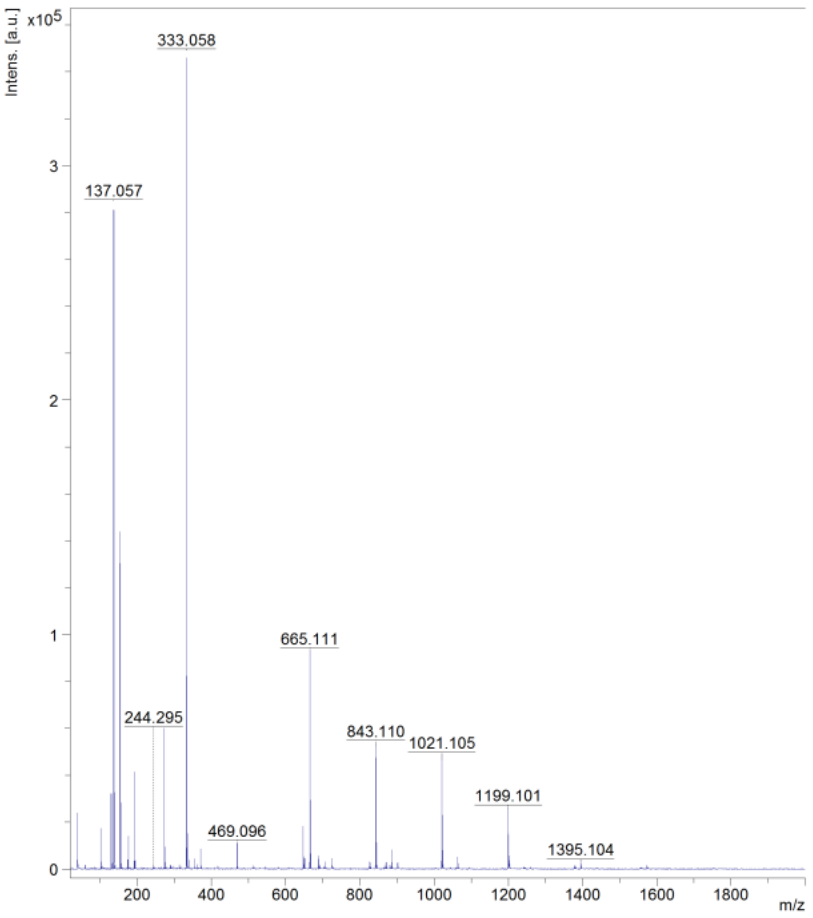

Figure S18. MALDI-TOF-MS spectrum of an oligomer of 2,3-dibromo-2-propen-1-ol, synthesized using coordination compound [VO(acac)$_2$(3-phenylpyridine)] and MAO as an activator (DHB matrix).

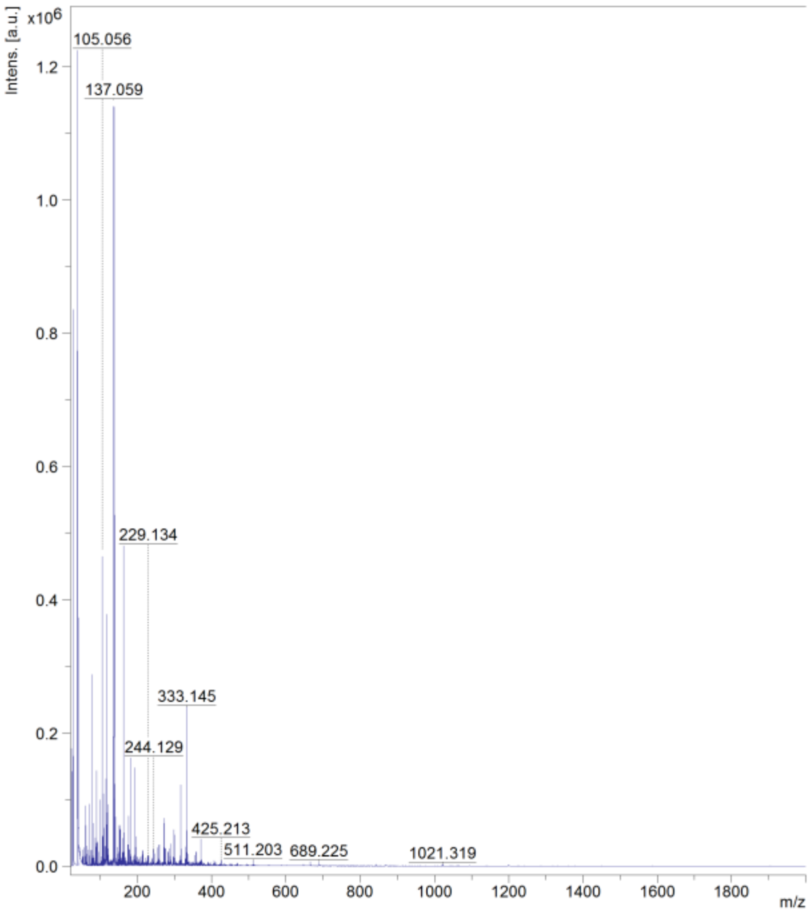

Figure S19. MALDI-TOF-MS spectrum of an oligomer of 2,3-dibromo-2-propen-1-ol, synthesized using coordination compound [VO(acac)$_2$(3-phenylpyridine)] and TMA as an activator (DHB matrix).

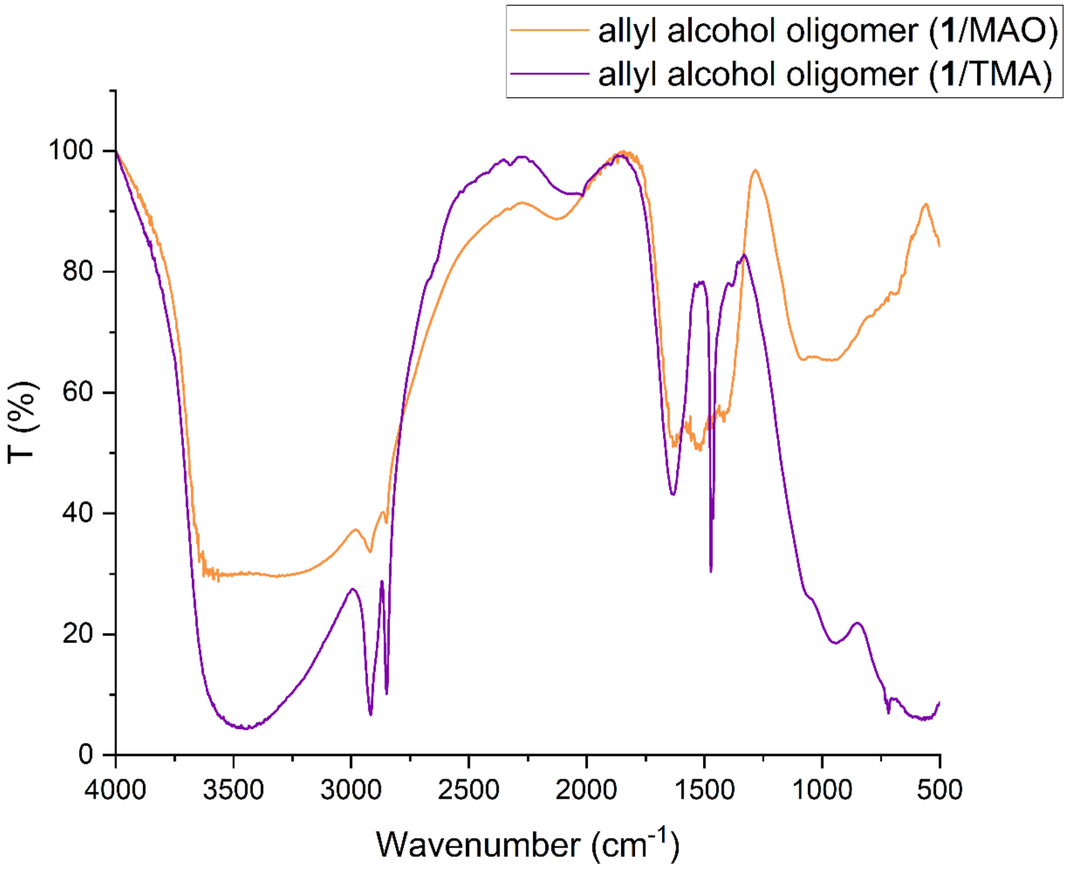

Figure S20. FT-IR spectrum of 2-propen-1-ol oligomer synthesized using coordination compound [VO(acac)$_2$(3-phenylpyridine)] and TMA/MAO.

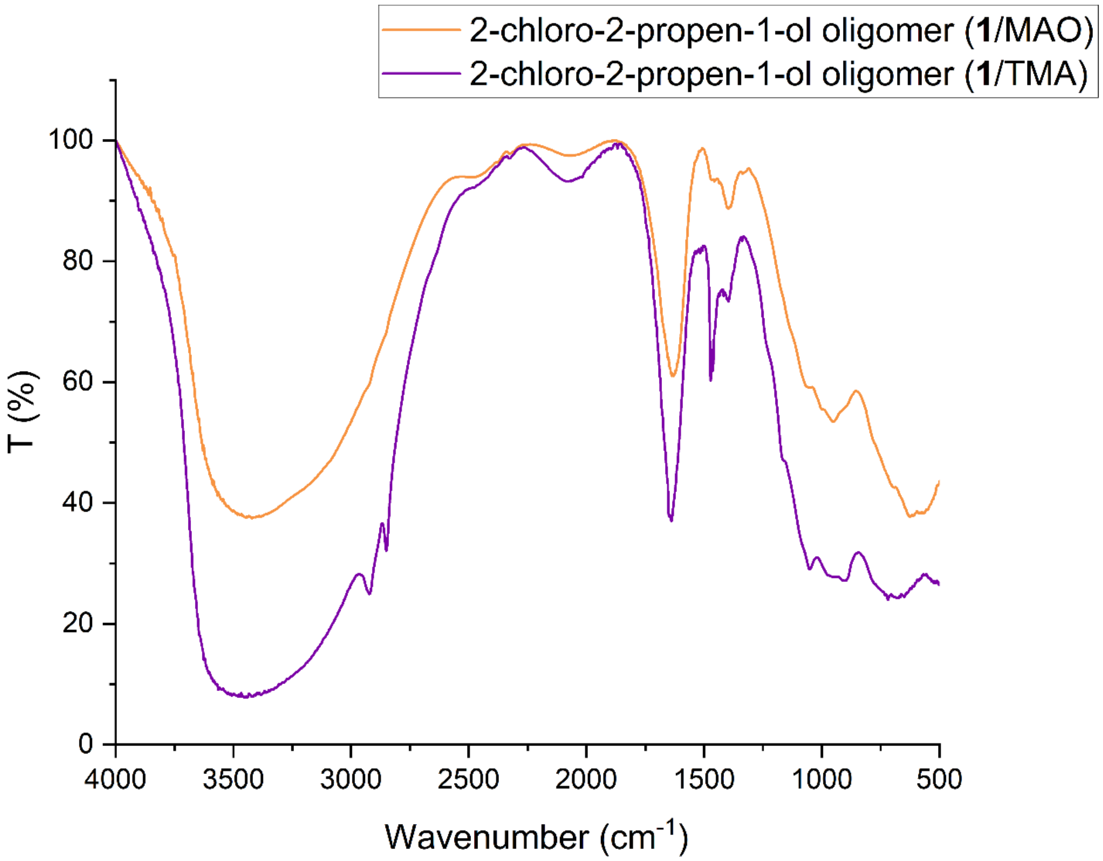

Figure S21. FT-IR spectrum of 2-chloro-2-propen-1-ol oligomer synthesized using coordination compound [VO(acac)$_2$(3-phenylpyridine)] and TMA/MAO.

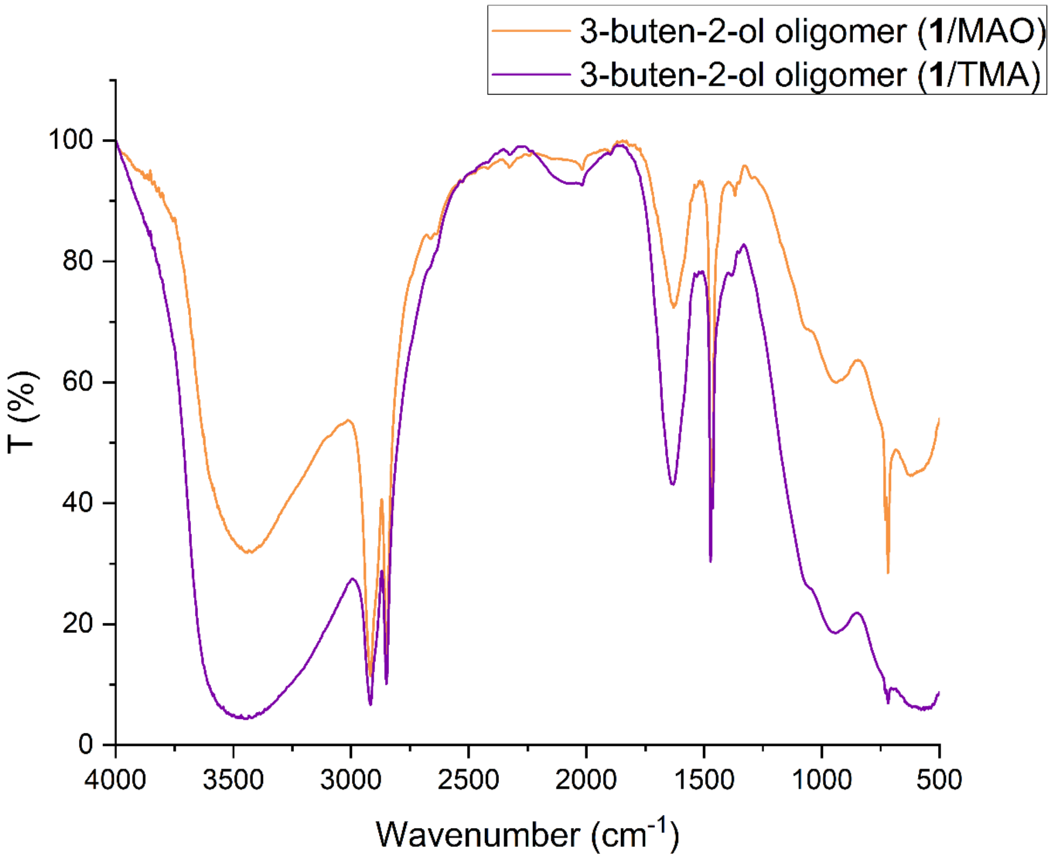

Figure S22. FT-IR spectrum of a 3-buten-2-ol oligomer synthesized using coordination compound [VO(acac)$_2$(3-phenylpyridine)] and TMA/MAO.

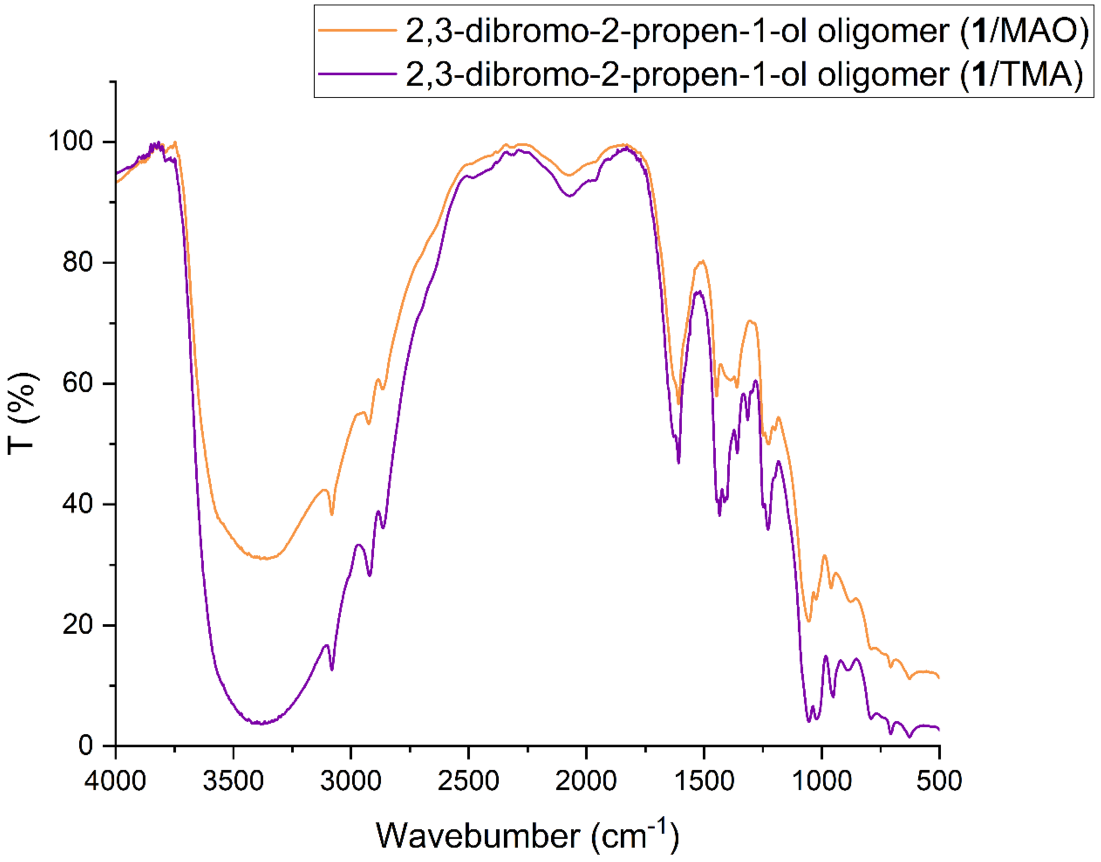

Figure S23. FT-IR spectrum of a 2,3-dibromo-2-propen-1-ol oligomer synthesized using coordination compound [VO(acac)$_2$(3-phenylpyridine)] and TMA/MAO.

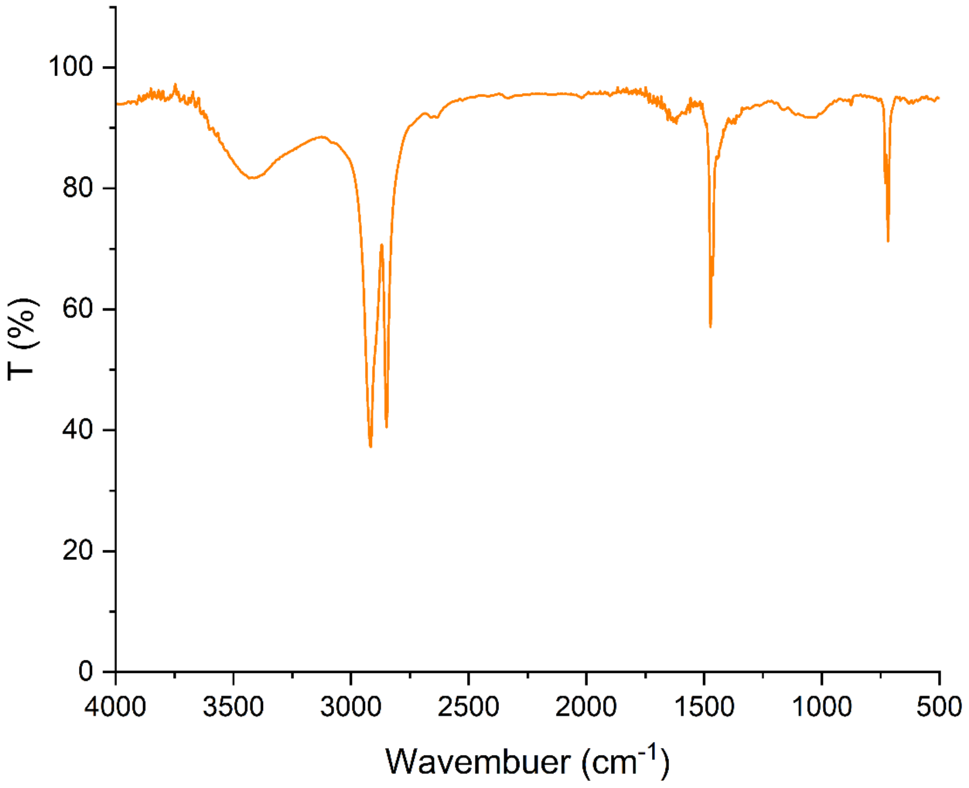

Figure S24. FT-IR spectrum of polyethylene synthesized using coordination compound [VO(acac)$_2$(3-phenylpyridine)] and Et_2AlCl as activator (sample D-3).

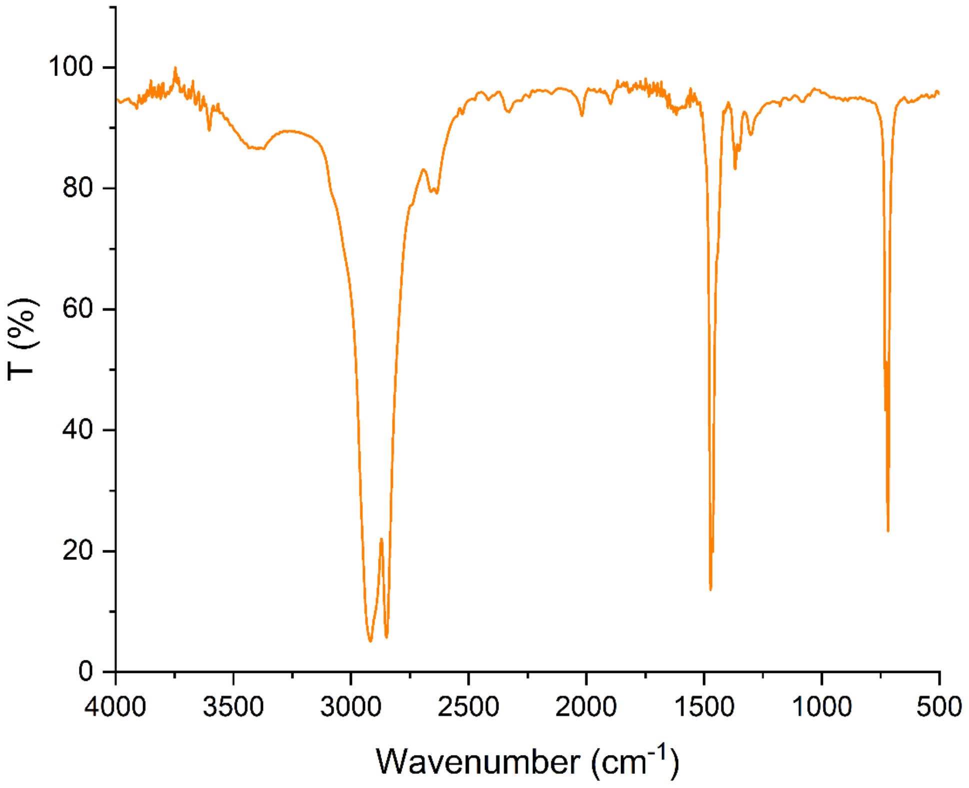

Figure S25. FT-IR spectrum of ethylene/1-octene copolymer synthesized using coordination compound [VO(acac)$_2$(3-phenylpyridine)] and Et_2AlCl as activator (sample D-6).

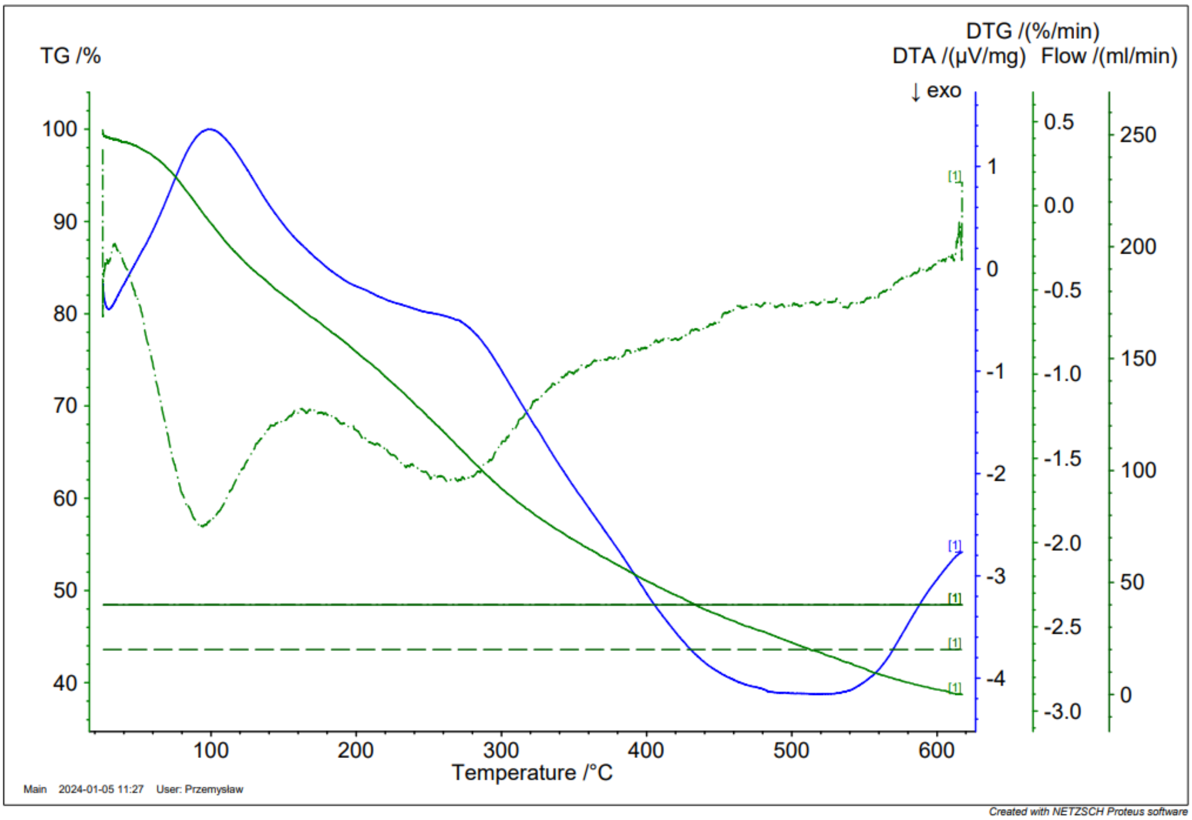

Figure S26. The TGA and DTA curves of 2-chloro-2-propen-2-ol oligomer obtained using [VO(acac)$_2$(3-phenylpyridine)]/MAO as a catalyst.

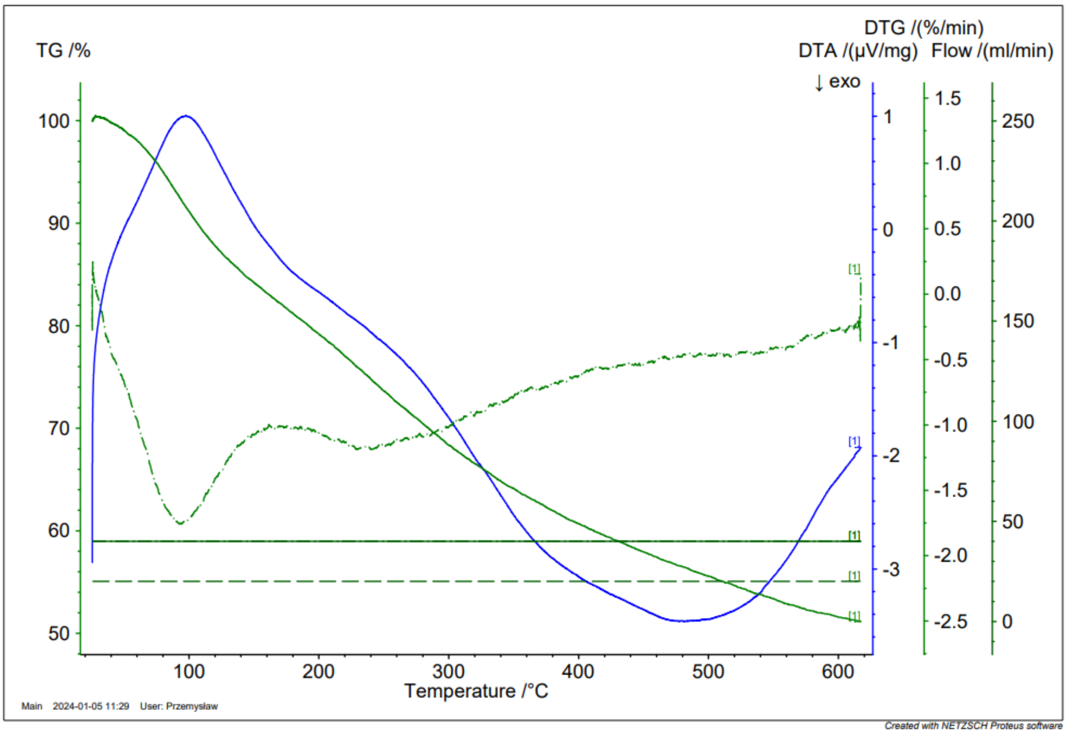

Figure S27. The TGA and DTA curves of 3-buten-2-ol oligomer obtained using [VO(acac)$_2$(3-phenylpyridine)]/MAO as a catalyst.

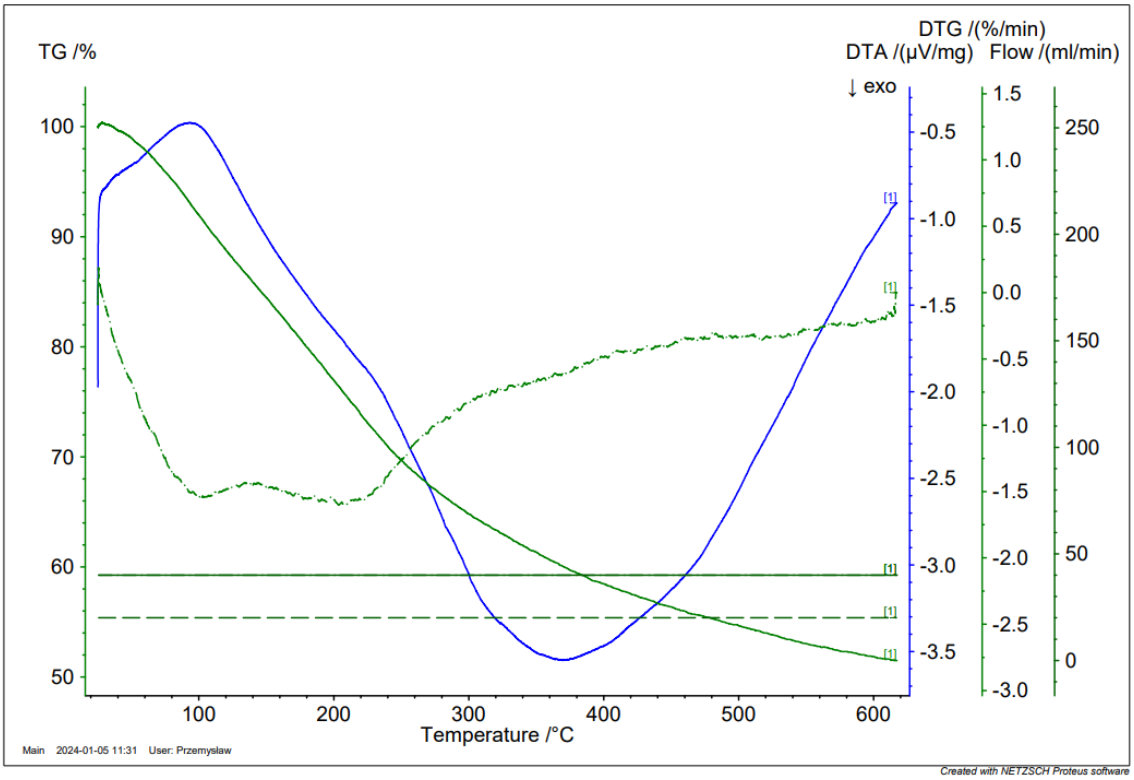

Figure S28. The TGA and DTA curves of 2-chloro-2-propen-1-ol oligomer obtained using [VO(acac)$_2$(3-phenylpyridine)]/TMA as a catalyst.

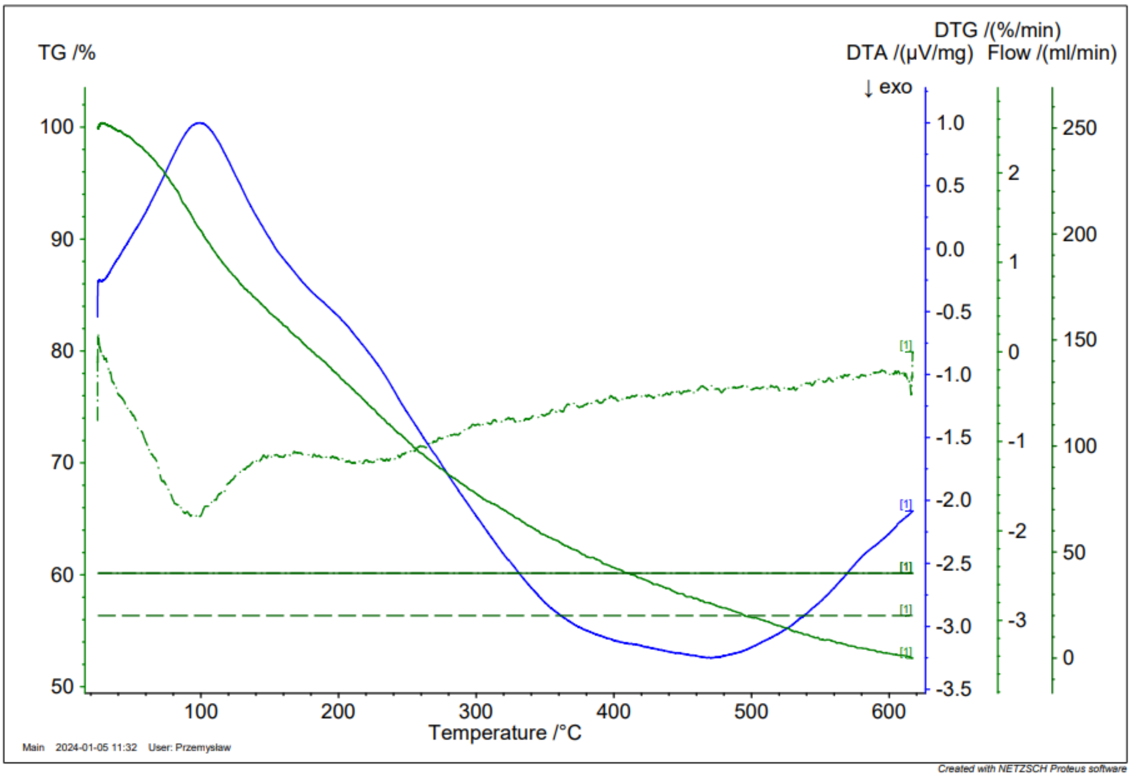

Figure S29. The TGA and DTA curves of 3-buten-2-ol oligomer obtained using [VO(acac)$_2$(3-phenylpyridine)]/TMA as a catalyst.

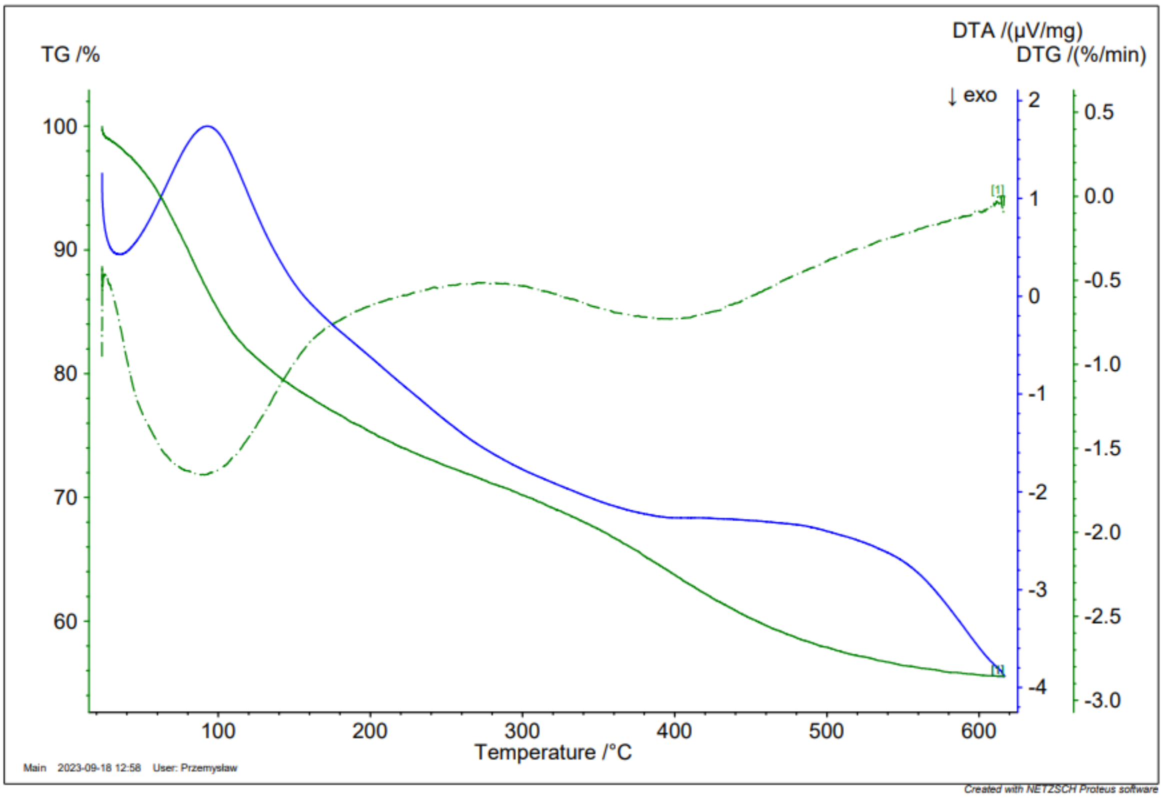

Figure S30. The TGA and DTA curves of 2-propen-1-ol oligomer obtained using [VO(acac)2(3-phenylpyridine)]/MAO as a catalyst.

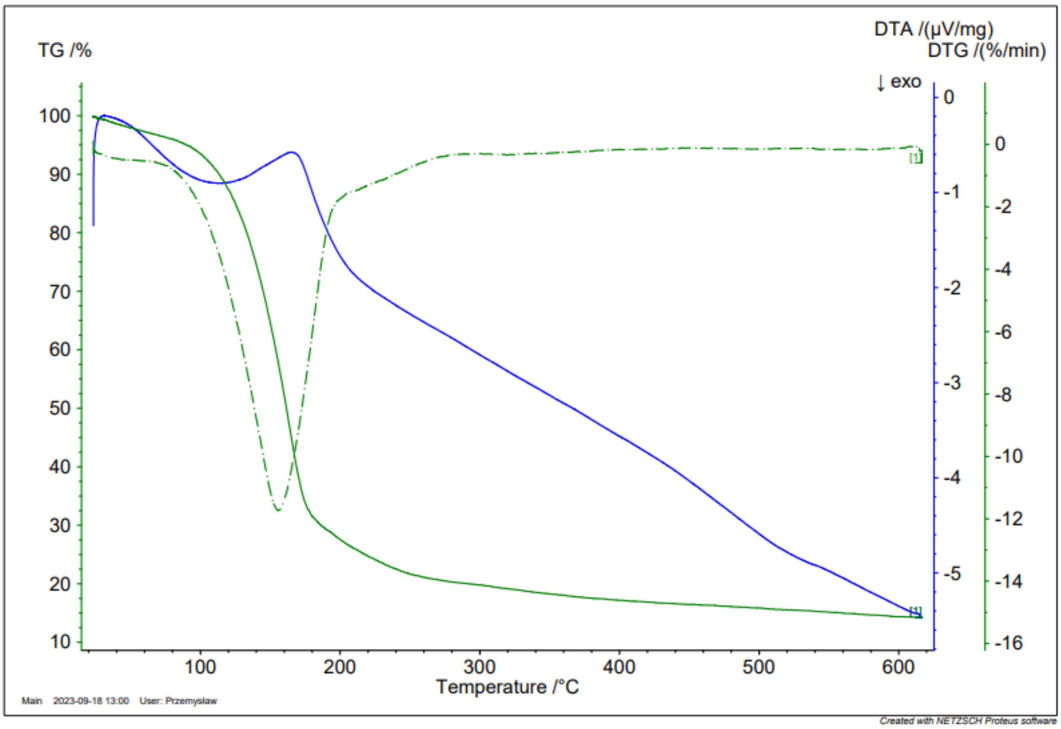

Figure S31. The TGA and DTA curves of 2,3-dibormo-2-propen-1-ol oligomer obtained using [VO(acac)2(3-phenylpyridine)]/MAO as a catalyst.

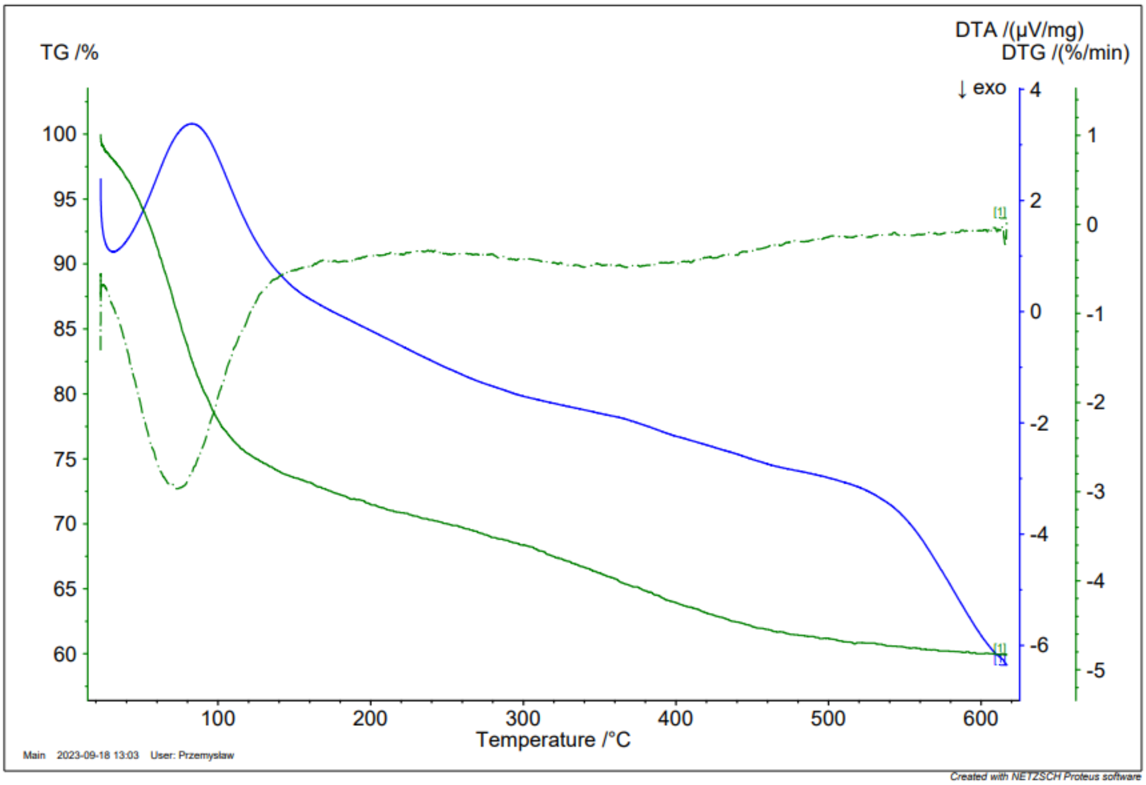

Figure S32. The TGA and DTA curves of 2-propen-1-ol oligomer obtained using [VO(acac)2(3-phenylpyridine)]/TMA as a catalyst.

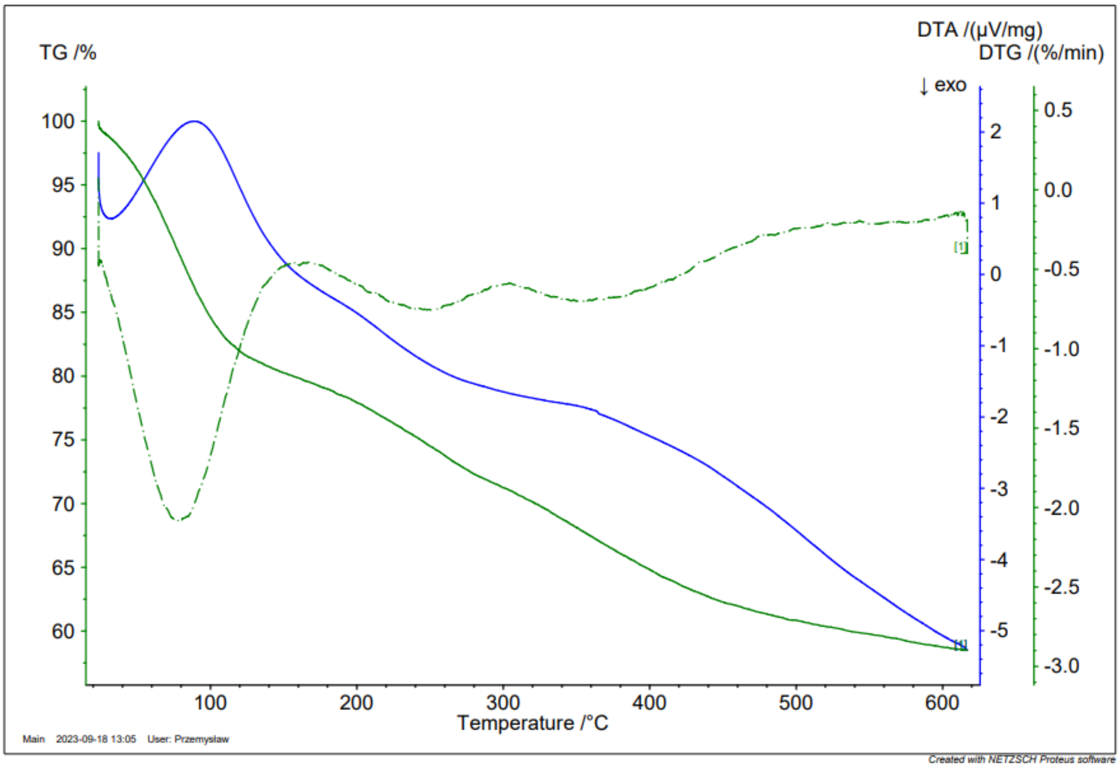

Figure S33. The TGA and DTA curves of 2,3-dibormo-2-propen-1-ol oligomer obtained using [VO(acac)2(3-phenylpyridine)]/TMA as a catalyst.

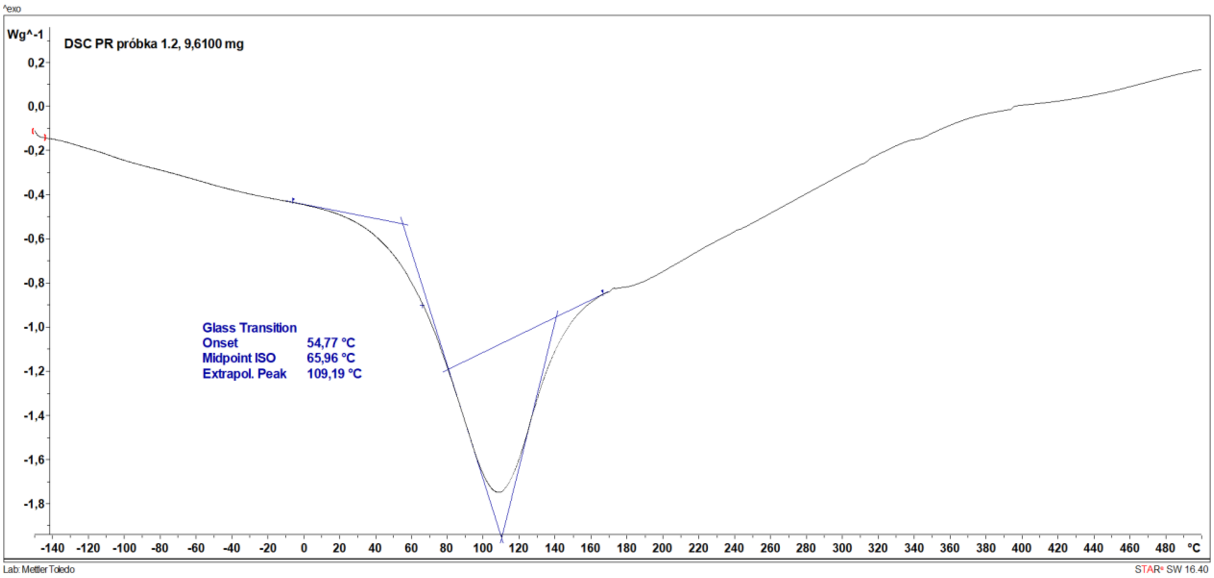

Figure S34. The DSC curve of 2-propen-1-ol oligomer obtained using [VO(acac)$_2$(3-phenylpyridine)]/MAO as a catalyst.

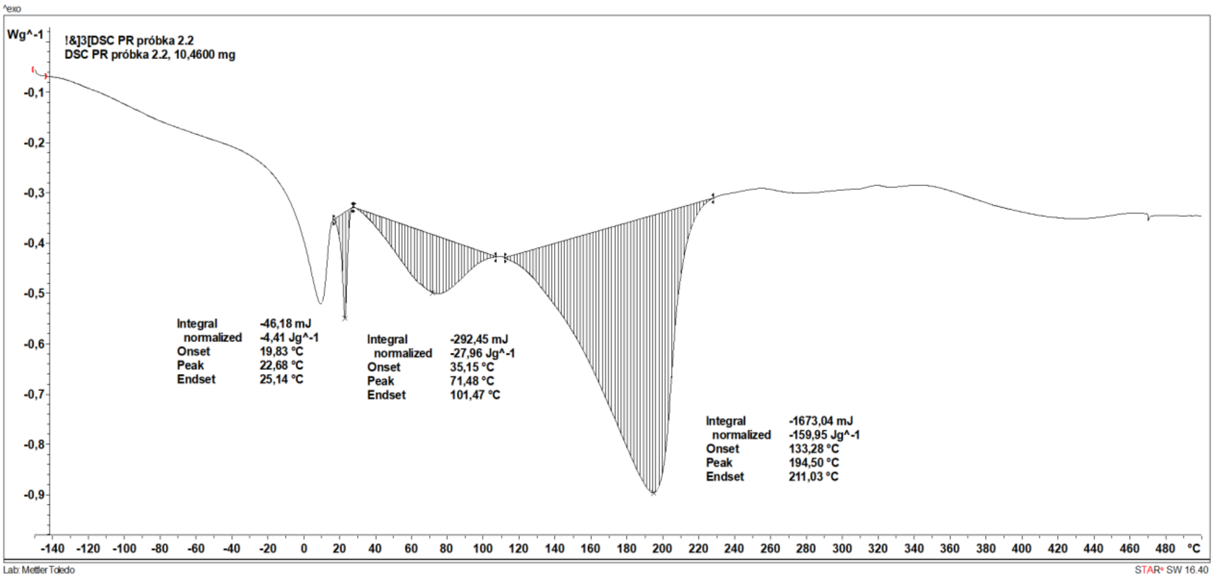

Figure S35. The DSC curve of 2,3-dibromo-2-propen-1-ol oligomer obtained using [VO(acac)$_2$(3-phenylpyridine)]/MAO as a catalyst.

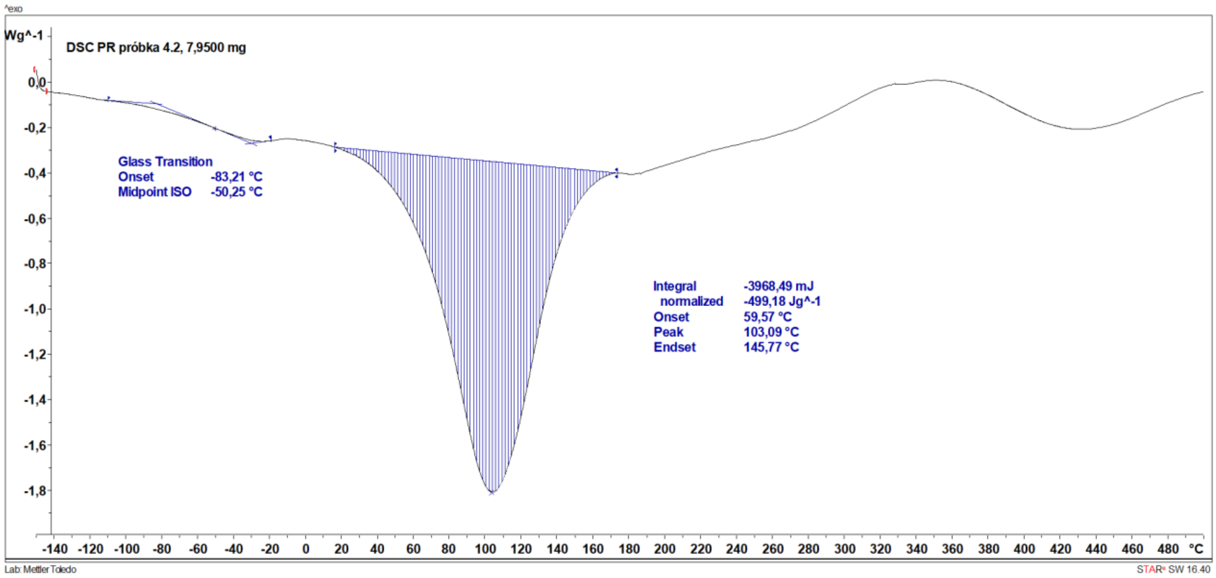

Figure S36. The DSC curve of 2-propen-1-ol oligomer obtained using $[VO(acac)_2$(3-phenylpyridine)]/TMA as a catalyst.

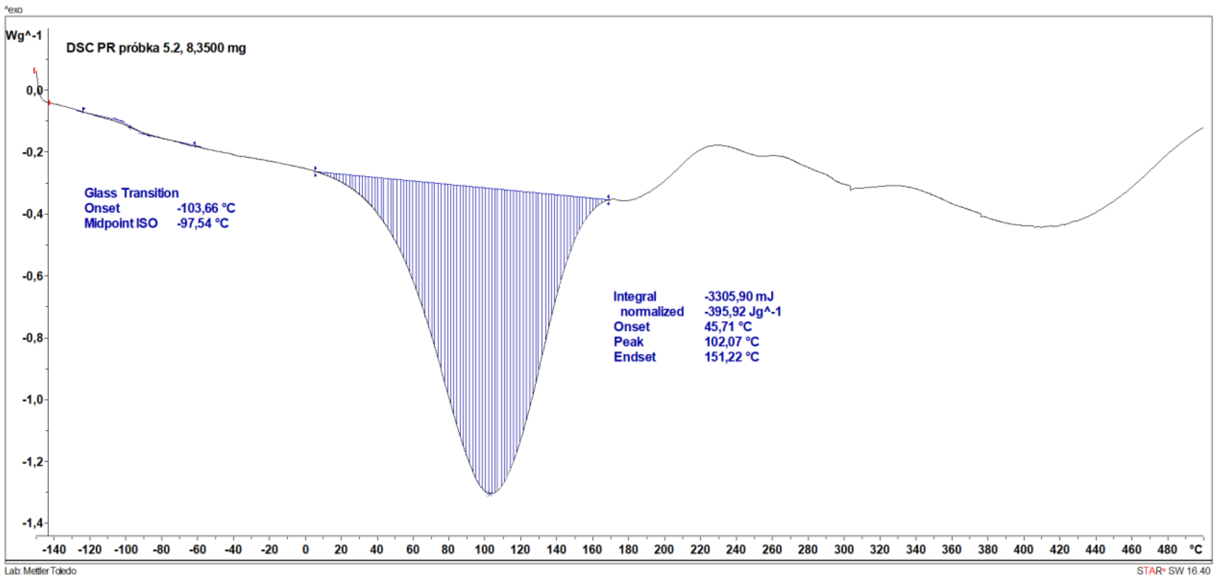

Figure S37. The DSC curve of 2,3-dibormo-2-propen-1-ol oligomer obtained using $[VO(acac)_2$(3-phenylpyridine)]/TMA as a catalyst.

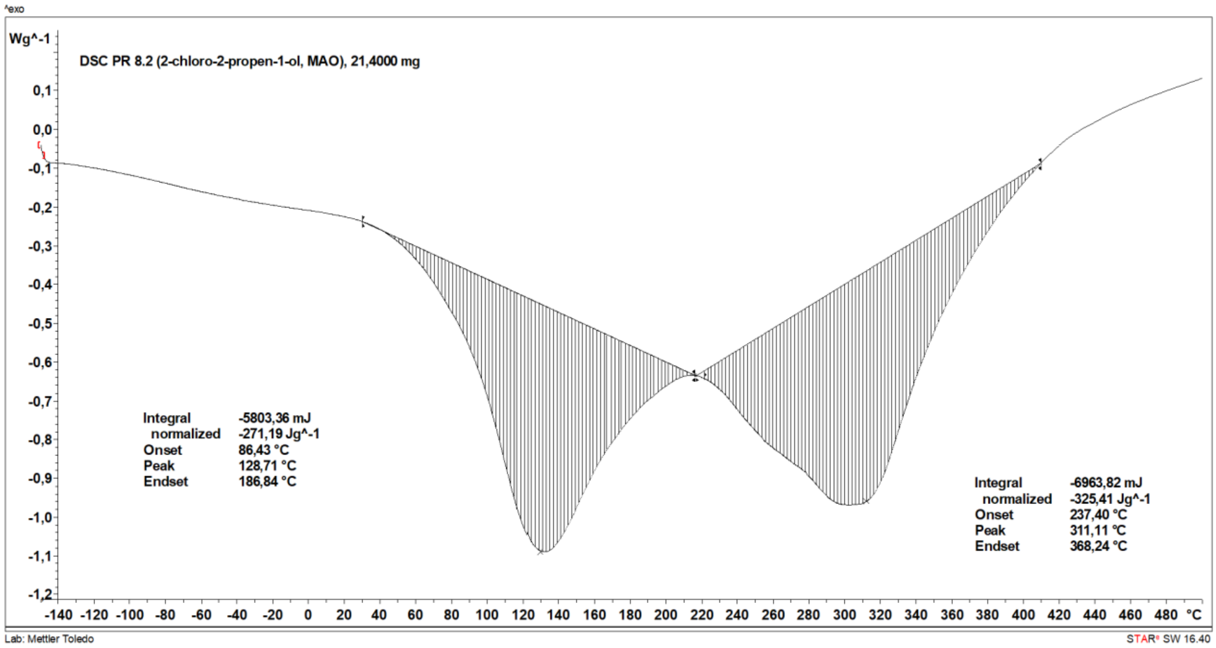

Figure S38. The DSC curve of 2-chloro-2-propen-1-ol oligomer obtained using [VO(acac)$_2$(3-phenylpyridine)]/MAO as a catalyst.

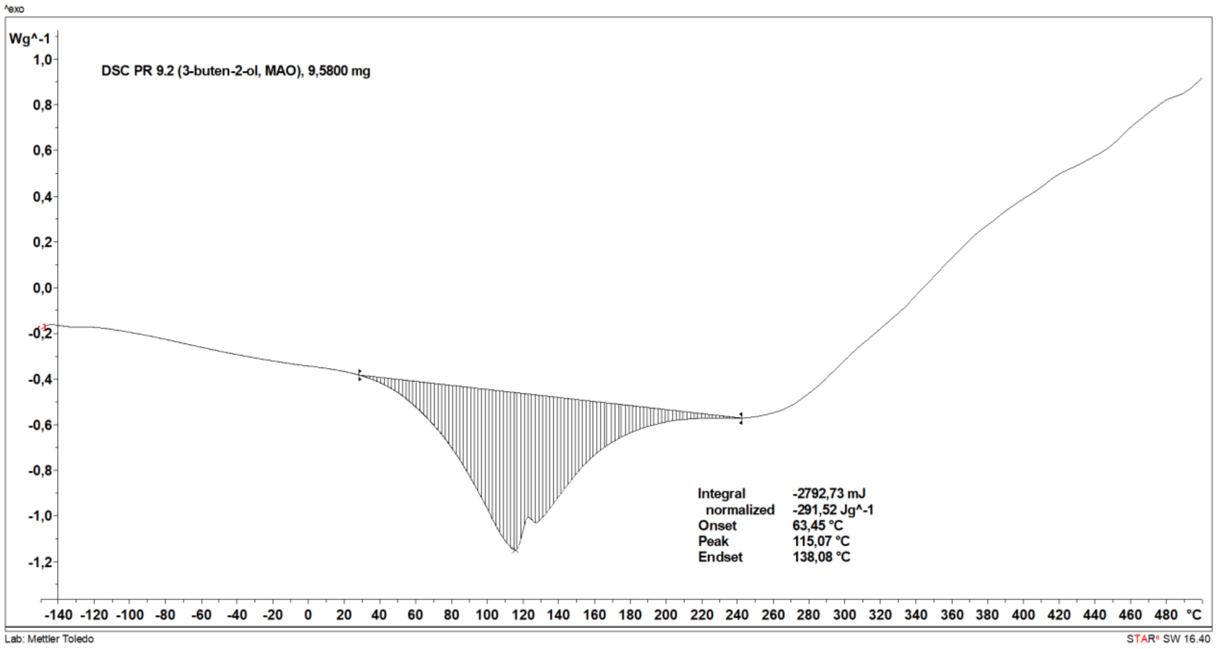

Figure S39. The DSC curve of 3-buten-2-ol oligomer obtained using [VO(acac)$_2$(3-phenylpyridine)]/MAO as a catalyst.

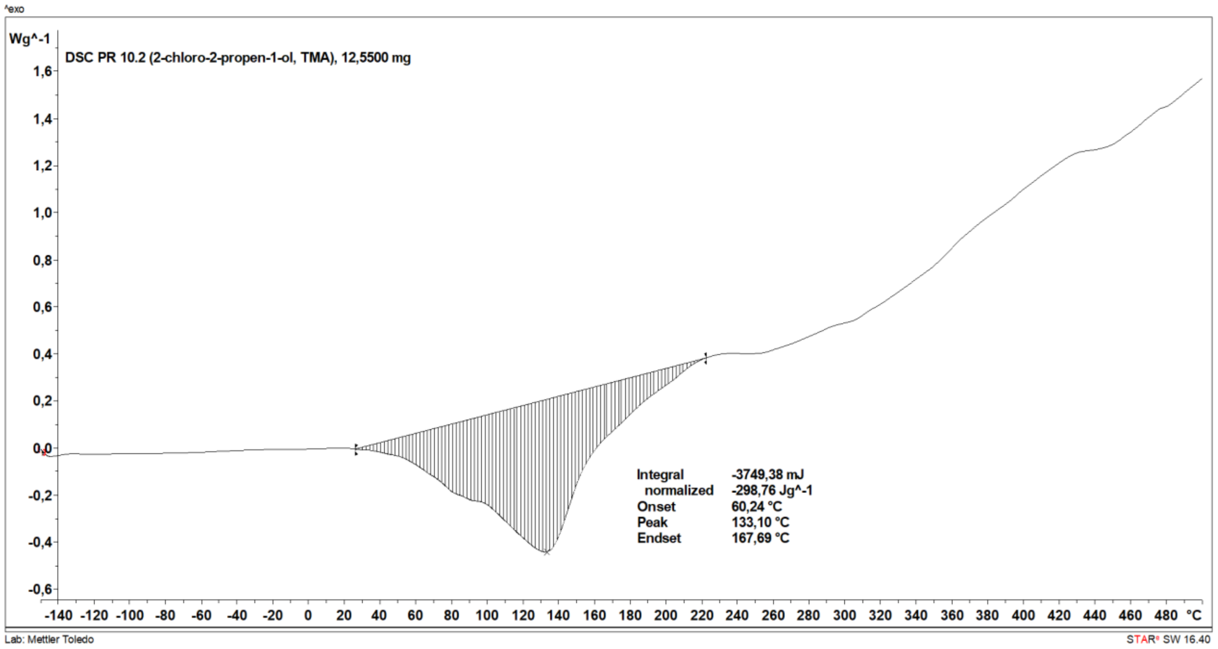

Figure S40. The DSC curve of 2-chloro-2-propen-1-ol oligomer obtained using [VO(acac)$_2$(3-phenylpyridine)]/TMA as a catalyst.

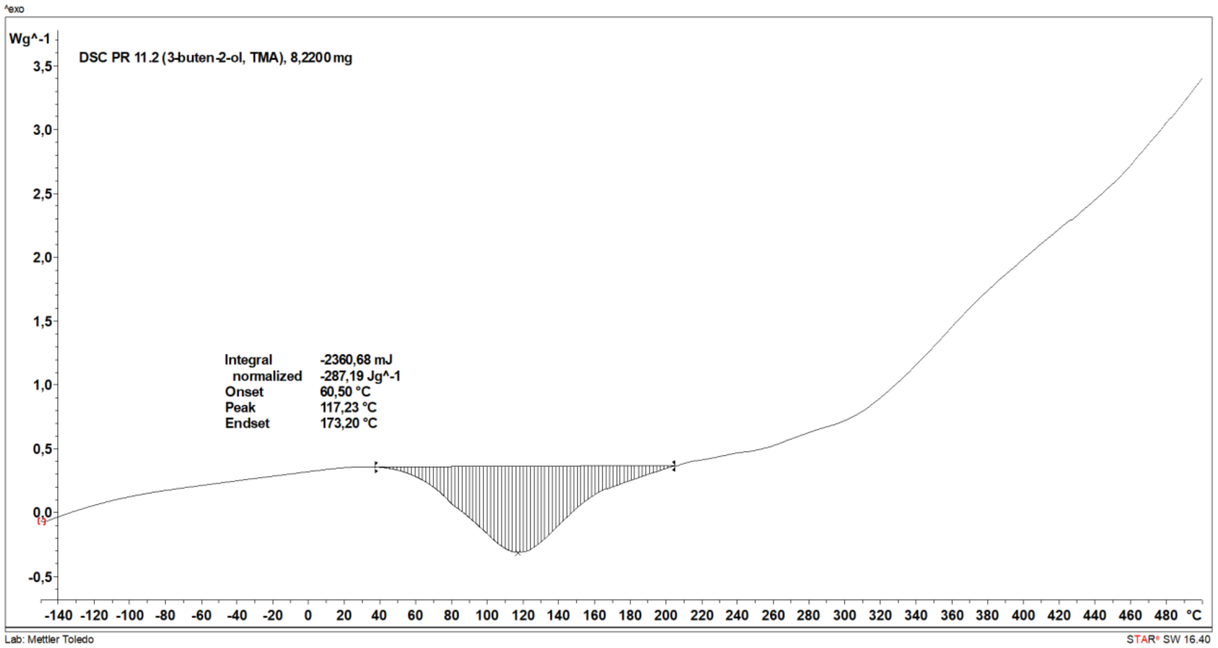

Figure S41. The DSC curve of 3-buten-2-ol oligomer obtained using [VO(acac)$_2$(3-phenylpyridine)]/TMA as a catalyst.

An increase in absorbance was observed already at higher wavelengths of approximately 350 nm when TMA was added as an activator. The absorbance maximum of *cis*/*trans*-[VO(acac)$_2$(3-ppy)] (**1**) at approximately 280 nm also increased with increasing TMA addition.

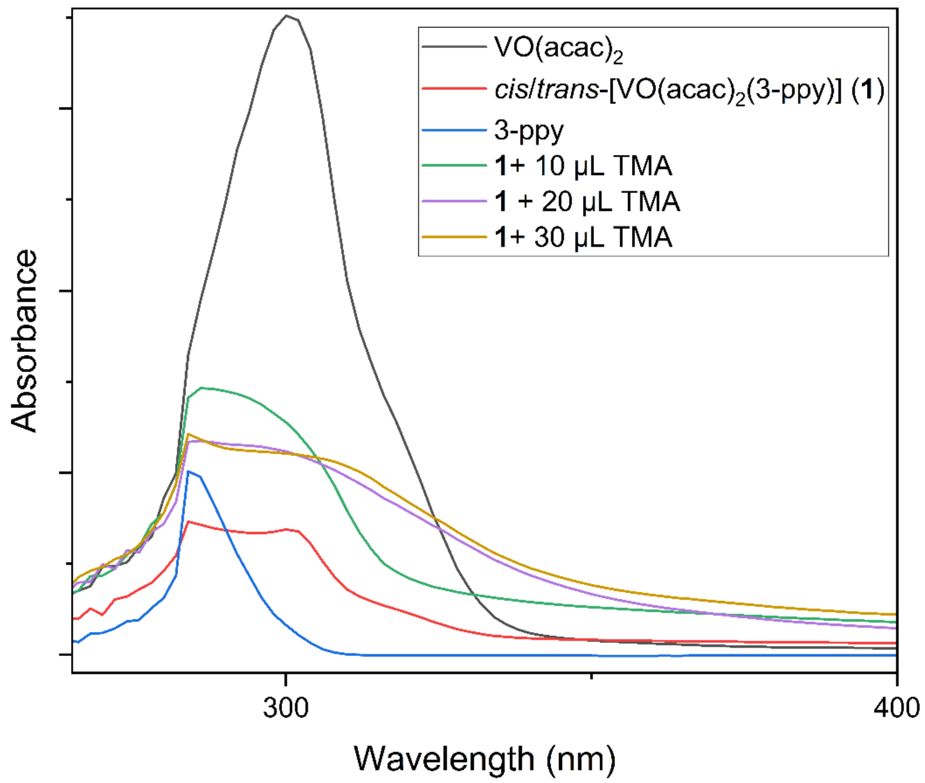

Figure S42. UV-VIS spectra of *cis*/*trans*-[VO(acac)$_2$(3-ppy)] (**1**), VO(acac)$_2$, 3-ppy, and *cis*/*trans*-[VO(acac)$_2$(3-ppy)] with TMA. All solutions were prepared in toluene: 0.5 mM VO(acac)$_2$, 0.5 mM 3-ppy, 0.5 mM *cis*/*trans*-[VO(acac)$_2$(3-ppy)].

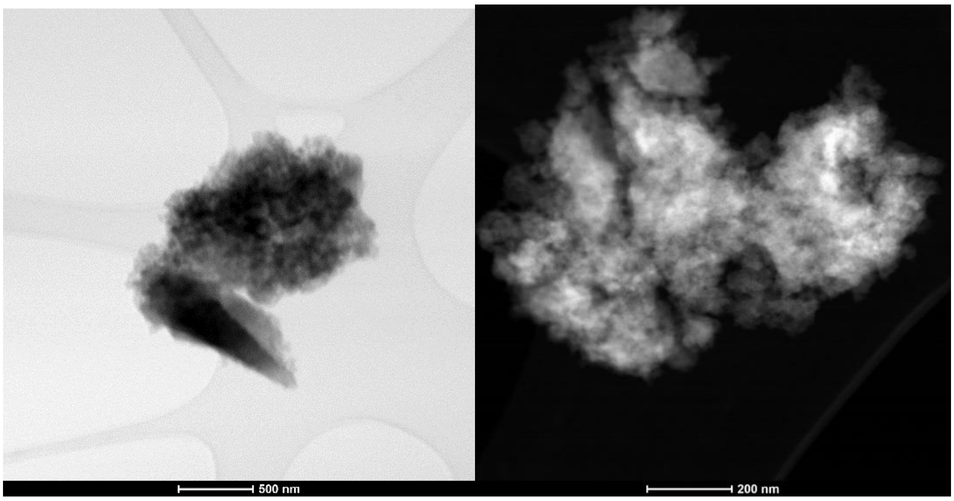

Figure S43. STEM image of oligo(3-buten-2-ol).

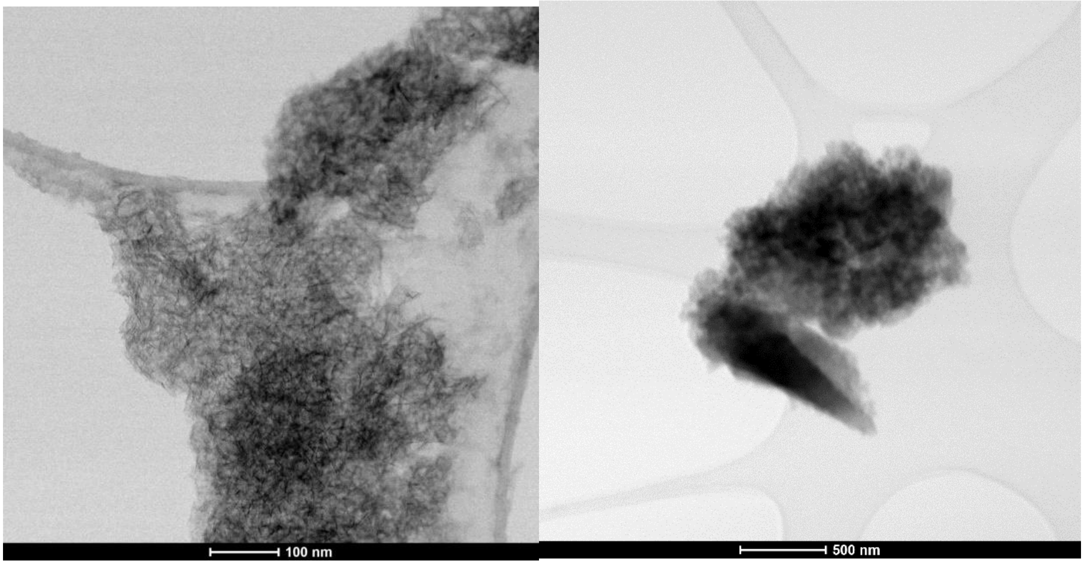

Figure S44. STEM image of oligo(2-chloro-2-propen-1-ol).

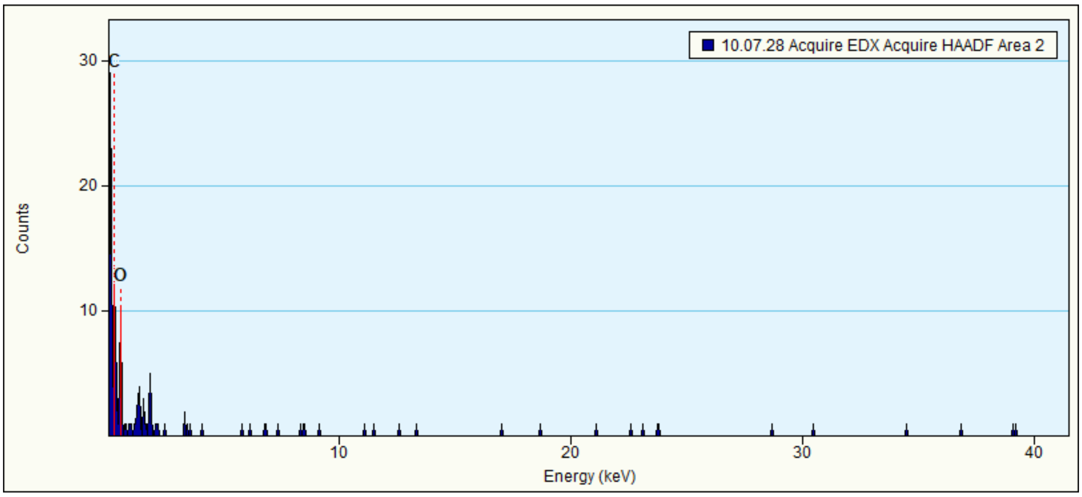

Figure S45. Energy-dispersive X-ray spectroscopy (EDX) measurements for oligo(3-buten-2-ol).

Table S3. Analysis of mass and atomic content performed by TEM.

Sample	Element	Weight %	Atomic %
oligo(3-buten-2-ol)	C	75.25	80.20
	O	24.74	19.79

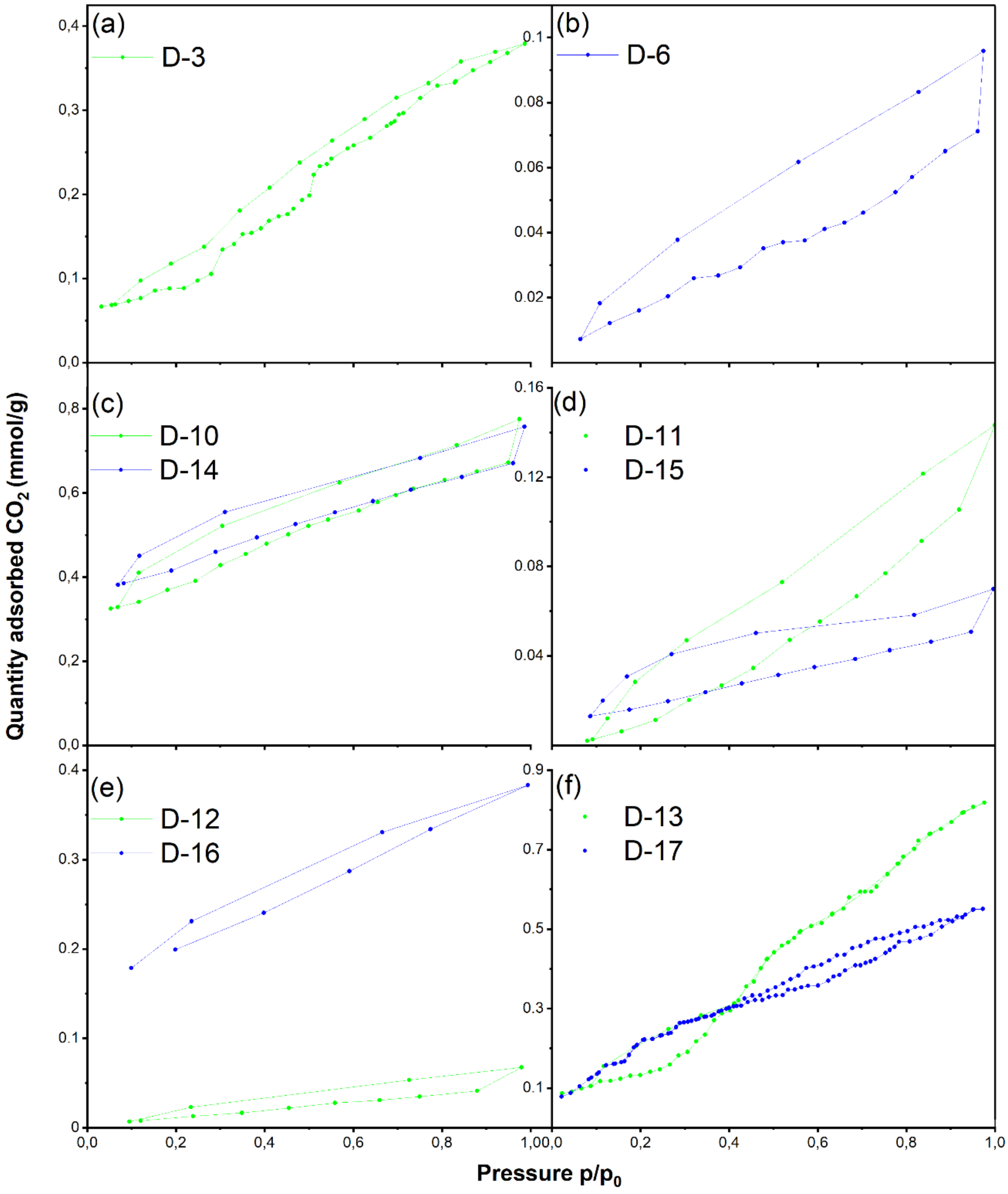

Figure S46. CO_2 adsorption and desorption isotherms of polymeric materials (a) polyethylene (D-3), (b) ethylene/1-octene copolymer (D-6), (c) 2-propen-1-ol oligomer synthesized using 1/MAO (D-11) and 1/TMA (D-14), (d) 2,3-dibromo-2-propen-1-ol oligomer synthesized using 1/MAO (D-11) and 1/TMA (D-16), (e) 2-chloro-2-propen-1-ol oligomer synthesized using 1/MAO (D-12) and 1/TMA (D-16), (f) 3-buten-2-ol oligomer synthesized using 1/MAO (D-13), 1/TMA (D-17).

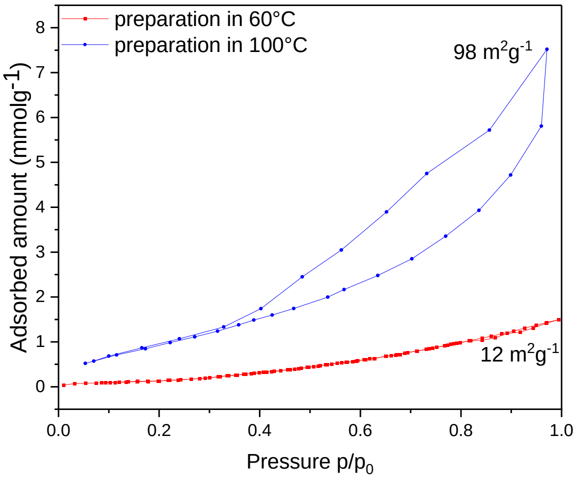

Figure S47. Comparison of adsorption and desorption isotherms of oligo(3-buten-2-ol) degassed at 60°C and 100°C .

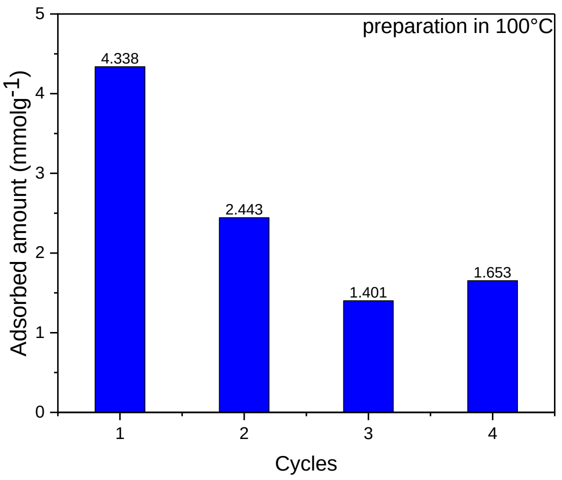

Figure S48. Stability of oligo(3-buten-2-ol) at 5 cycles. Sample degassed in 100°C.

Table S4. Catalytic activity of vanadium(IV) coordination compounds in the ethylene polymerization reaction.

Coordination complex compund	Cocatalyst	ETA/V	Al/V	T (°C)	p[a] (bar)	Catalytic activity[b]	Ref.
[VO(dipic)]	$EtAlCl_2$	200	1000	60	5	613	[1]
[VO(dipic)(bipy)]	$EtAlCl_2$	200	1000	60	5	11040	[1]
[VO(dipic)(phen)]	$EtAlCl_2$	200	1000	60	5	2726	[1]
[VO(tda)(bipy)]	MMAO-12	-	1000	50	0.5	555.56	[2]
[VO(ida)(bipy)]	MMAO-12	-	1000	50	0.5	738.9	[2]
[VO(oda)(bipy)]	MMAO-12	-	1000	50	0.5	366.7	[2]
[VO(tda)]	MMAO-12	-	1000	50	0.5	476.2	[2]
[VO(ida)(phen)]	MMAO-12	-	1000	50	0.5	396.55	[2]
[VO(acacen)]	$EtAlCl_2$	-	129	30	5	534.2	[3]
[VO(acetph)]	$EtAlCl_2$	-	129	30	5	156.6	[3]
$[V({=}N)(2,6\text{-}Cl_2\text{-}C_6H_3)Cl_2(PMe_2Ph)_2]$	Et_2AlCl	10	500	20	1.01	2072	[4]
$[V({=}N)(2,6\text{-}{}^iPr_2\text{-}C_6H_3)Cl_2(PMe_2Ph)_2]$	Et_2AlCl	10	500	20	1.01	2168	[4]
$[V({=}N)({}^tBu)Cl_2(PMe_2Ph)_2]$	Et_2AlCl	10	500	20	1.01	1956	[4]
$[V\{Me_2NCH_2CH_2N(CH_2\text{-}2\text{-}O\text{-}3,5\text{-}{}^tBu_2\text{-}C_6H_2)_2\}Cl_2$	$EtAlCl_2$	-	5000	30	5	5500	[5]
$[V\{Me_2NCH_2CH_2N(CH_2\text{-}2\text{-}O\text{-}3,5\text{-}{}^tBu_2\text{-}C_6H_2)(CH_2\text{-}2\text{-}O\text{-}C_6H_4)\}Cl_2]$	$EtAlCl_2$	-	50	30	5	60	[5]
$VO(acac)_2$	$Et_2AlCl/Mg(C_4H_9)_2$	-	275	30	2.8	123	[6]
$[VO(acac)_2$(3-phenylpyridine)]	Et_2AlCl	200	3000	60	5	83400	This study

[a]pressure of ethylene, [b]Catalytic activity in the polymerization reaction of ethylene [kg/(molv·h)], dipic = dipicolinate anion, bipy = 2,2′-bipyridine, ida = iminodiacetate anion, phen = 1,10-phenatroline, oda = diglycolate anion , tda = thiodiacetate anion, H_2acacen = N,N′-ethylenebis(acetylacetonimine), H_2acetf = N,N′-phenylene-1,2-bis(2-hydroxyacetophenoneimine), Ph = phenyl group, Me = methyl group, ^{i}Pr = isopropyl group, ^{t}Bu = *tert*-butyl group, acac = acetylacetone, MMAO-12 = modified methylaluminoxane.

Funding

Publication financed from the state budget under the program of the Ministry of Education and Science entitled Pearls of Science project no. PN/01/0137/2022, grant amount 239,800.00 PLN, total project value 239,800.00 PLN (Poland).

Acknowledgements

This publication is the subject filed with the Patent Office (application number P.448299).

The in-house X-ray diffraction experiments were carried out at the Department of Physics, University of Warsaw, on a Rigaku Oxford Diffraction SuperNova diffractometer, which was co-financed by the European Union within the European Regional Development Fund (POIG.02.01.00-14-122/09).

References

[1] J. Drzeżdżon, M. Białek, P. Parnicka, A. Zaleska-Medynska, *ChemistrySelect* **2024**, *9*, DOI 10.1002/slct.202303255.

[2] M. Pawlak, J. Drzeżdżon, B. Gawdzik, A. Gołąbiewska, P. Mielczarek, A. Nowak, Z. Wzorek, D. Jacewicz, *Polyhedron* **2024**, *250*, 116810.

[3] M. Białek, A. Leksza, A. Piechota, K. Kurzak, K. Koprek, *Journal of Polymer Research* **2014**, *21*, 389.

[4] G. Zanchin, L. Vendier, I. Pierro, F. Bertini, G. Ricci, C. Lorber, G. Leone, *Organometallics* **2018**, *37*, 3181–3195.

[5] M. Białek, E. Bisz, *J Catal* **2018**, *362*, 65–73.

[6] L. A. Rishina, Y. V Kissin, S. S. Lalayan, P. M. Nedorezova, V. G. Krasheninnikov, *Polym Int* **2022**, *71*, 338–347.
